# Bioactive, antioxidant and antimicrobial properties of chemically fingerprinted essential oil*s* extracted from *Eucalyptus globulus*: *in-vitro* and *in-silico* investigations

**DOI:** 10.3389/fchem.2023.1287317

**Published:** 2023-12-08

**Authors:** Said Nasir Shah, Ibrar Khan, Sidra Tul Muntaha, Azam Hayat, Mujaddad Ur Rehman, Tawaf Ali Shah, Farhan Siddique, Ahmad Mohammad Salamatullah, Amare Bitew Mekonnen, Mohammed Bourhia

**Affiliations:** ^1^ Department of Microbiology, Abbottabad University of Science & Technology, Abbottabad, Pakistan; ^2^ Department of Zoology, Abbottabad University of Science & Technology, Abbottabad, Pakistan; ^3^ Department of Biotechnology, University of Okara, Okara, Pakistan; ^4^ Laboratory of Organic Electronics, Department of Science and Technology, Linköping University, Linköping, Sweden; ^5^ Department of Food Science and Nutrition, College of Food and Agricultural Sciences, King Saud University, Riyadh, Saudi Arabia; ^6^ Department of Biology, Bahir Dar University, Bahir Dar, Ethiopia; ^7^ Laboratory of Chemistry and Biochemistry, Faculty of Medicine and Pharmacy, Ibn Zohr University, Agadir, Morocco

**Keywords:** essential oils, *Eucalyptus*, disinfectants, GC-MS, molecular docking

## Abstract

Innovative approaches are urgently required to treat divestating bacterial diseases in the face of rising bacterial resistance rates. The current investigative work focused on hydro-distilling Tasmanian blue gum (*Eucalyptus globulus*) to isolate the essential oil, which was then tested for bioactivity, antioxidant capacity, and antibacterial activity using *in-vitro* and *in silico* assays. The antioxidant activity was avualated against DPPH and FRAP. With results of 69.63 RSA (%) (µL/L AAE) at a concentration of 5 mL/L and 51.56 (µL/L AAE) at concentration of 90 ppm in the 2,20-diphenyl-1-picryl hydrazyl (DPPH) and ferric reducing antioxidant power (FRAP) assays, respectively, the extracted oil indicated considerable antioxidant activity. The extracted oil demonstrated powerful antibacterial activity in *in-vitro* tests against both Gram-positive and Gram-negative bacterial strains, including *Bordetella bronchiseptica* (21 mm), *Staphylococcus epidermidis* (19 mm), and *Staphylococcus aureus* (19 mm), with significant minimal inhibitory (MIC) and minimum bactericidal (MIB) concentrations. Additionally, GC-MS analysis of the oil from E. globulus identified several low-molecular-weight compounds, including Eucalyptol, γ-Terpinene, Shisool acetate, 1,3-trans,5-cis-Octatriene, 2,6-Dimethyl-1,3,5,7-octatetraene, E,E, Cyclohexene, 1-methyl-4-(1-methylethylidene), Benzene, 1-methyl-4-(1-methylethenyl), Butanoic acid, 3-methyl-, 3-methylbutyl ester, and 1,3,8-p-Menthatriene. Several other compounds were also identified, including Fenchol, 2-Methyl-trans-3a,4,7,7a-tetrahydroindane, (E,E,E)-2,4,6-Octatriene, 1,2,3,6-Tetrahydrobenzylalcohol, acetate, Alloaromadendrene, Phenol, 2-ethyl-4,5-dimethyl, Phenol, 2-methyl-5-(1-methylethyl)-, p-Cymen-7-ol, 1,5,5-Trimethyl-6-methylene-cyclohexene, 1,3-Cyclohexadiene, 1-methyl-4-(1-methylethyl)-, 2,6-Octadien-1-ol, 3,7-dimethyl-, acetate, (Z), and more. The bioactive potential of *Eucalyptus globulus* essential oil against 1AJ6 and 1R4U was highlighted by molecular docking analyses, suggesting its utility as a natural source of antioxidant and antibacterial compounds with the potential to replace chemical disinfectants in a variety of applications.

## 1 Introduction

Traditional Herbal medicine has always been the cheapest and easiest way to treat people in primary healthcare systems in places with few resources. Local people have been using plants as medicine for a long time. Fossils showed that people have been using plants as medicine since the Middle Paleolithic era, which gives them an edge here (often hundreds or thousands of years) (Rafat et al., 2010). Since the beginning, people have used plants as medicine to treat illnesses in the ensuing years (Prakash and Gupta, 2005). Indigenous healthcare systems worldwide use medicinal plants as essential to their care (Farnsworth, 1990). Most things sold as “traditional herbal remedies” have been around for a long time, and many herbal medicines on the market today are the same way. Even though modern medicine and these old-fashioned methods can work together, herbal treatments have often stayed popular (Vishwakarma et al., 2013). In the past few years, it has become more common to use knowledge from traditional medicine to study plants (Jones et al., 2006).

Several secondary metabolites produced by medicinal plants have potent antibacterial properties, which have long been used in traditional medicine. According to research, some herbs have been shown to help with skin cleaning. Processed extracts of these plants are often used to scrub the skin. *Eucalyptus robusta* and *Aloe barbadensis* are two of the most often utilized plants for disinfection in the home. For respiratory infections, *Andrographis paniculata*, *Berberis vulgaris,* and *Cinnamomum verum* are among the most often utilized herbs as antimicrobial and antiseptics (Dixit and Mittal, 2013). Various germ-killing plants were tested in research on herbal hand washes and sanitizers Tests of polyherbal handwashes produced and tested to fight *Putida vulgaris* and other skin illnesses discovered that they were all ineffective against these bacteria (Saad et al., 2011). There have been reports of bacteriostatic effects on hands and inanimate objects for a variety of plant mixtures. Using herbal hand sanitizer, *Coleus vettiveroides, Coriandrum sativum* and *Citrus limon, Vetiveria zizaniides,* and *Azadirachta indica* extracts were shown to be safe, effective, and had high antibacterial and cooling properties. Compared to the reference product, the herbal hand soap proved to be efficient and safe against *E. coli, Pseudomonas aeuroginosa, Staphylococcus aureus, Bacillus subtilis, Saccharomyces cerevisie,* and *Candida albicans* (Kadapure et al., 2022). Herbal toothpowder was also found to be as effective as commercial brands including Colgate and Pepsodent (Vohra et al., 2012). Plants that are aromatic and medicinal produce secondary compounds such as terpenoids, alcoholic compounds (such as geraniol and menthol), aldehydes (like citral and carvone camphor), acidic compounds (like benzoic acid, cinnamic acid, and myristic acid), aldehydes (like citral and carvone camphor), and ketonic bodies (such as thymol) that are used in aromatherapy (e.g., ascaridole, anethole) (Koul et al., 2008).

Since ancient times, the *Eucalyptus* tree has been used as an ethnomedicinal plant and is currently one of the most frequently planted genera in the Myrtaceae. This is a fast-growing tree that will thrive in any environment. Flu, fever, sore throat, respiratory tract infections, urinary tract infections, and bronchial infections may be treated with eucalyptus trees. The species’ anti-cancer properties have also been shown (Damjanović-Vratnica et al., 2011). As a result, *Eucalyptus* spp. Essential oil is widely used all over the globe. Since this achievement, the utilization of these chemicals as raw materials in the production of food, medicine, and cosmetics has expanded significantly (Fabiyi et al., 2020). A large variety of foods and drinks include eucalyptol, which is utilized in flavoring, scenting, and cosmetics as well, as well (Harkat-Madouri et al., 2015). Oil from eucalyptus leaves may be used to treat a variety of ailments. This ingredient might be used in dental care products due to its anti-inflammatory and antifungal properties, as well as its insecticide and acaricidal actions (Vigo et al., 2004). The Widespread usage of essential oil of medicinal plants as food and beverage flavorings, medicinal perfumes, and other product scenting agents attests to the versatility of their aroma distributed globally (Clark, 1996).

The World Health Organization (WHO) strategically incorporates the use of plant medicines in accordance with established conventional medical practices. The cultivation and processing of medicinal plants, utilizing established agro-industrial techniques, are of utmost significance in the manufacturing of herbal medicines. It is worth noting that modern pharmaceuticals frequently utilize components derived from medicinal plants (Newman and Cragg, 2007). Considering the increasing prevalence of bacterial resistance, there is a need for innovative interventions, despite the availability of established and well-validated methods for mitigating bacterial infections. Within this particular context, herbal medicine presents itself as a promising avenue for addressing pathogenic bacteria, showcasing potential opportunities for commercial utilization.

Natural goods are far more important than synthetic equivalents because of their inherent biological compatibility, lower toxicity, and wider range of bioactivity. Additionally, they support sustainability issues, provide a variety of solutions across different industries, and meet the market’s rising desire for natural and organic goods (Yadav et al., 2022). Furthermore, the *in silico* methodology is a cutting-edge and environmentally friendly way to assess the biological potential of bioactive substances derived from plants. It eliminates the need for time-consuming lab studies by using computational algorithms to predict pharmacological qualities quickly and affordably. This eco-conscious methodology is a useful tool for rapidly investigating bioactive compounds in plant-based medicine and other sectors because it not only speeds up drug discovery but also helps with compound design optimization (Aldossari et al., 2023; Yusuf, 2023).

Taking into consideration the existing challenges, the objective of this study is to utilize the inherent properties of medicinal plants to effectively combat bacterial infections. This research aims to isolate essential oils with therapeutic potential, specifically focusing on *Eucalyptus globulus*. The study aims to utilize the antimicrobial properties of these essential oils to make a valuable contribution to the advancement of alternative disinfectants and antiseptic agents. The investigation focuses on enhancing the interaction between alcohol and phenolic compounds, taking advantage of their volatility and reactivity, to produce effective formulations with minimal contact time. The objective of this study is to develop a new methodology based on the utilization of indigenous medicinal plants, with the aim of providing a potential solution for combating bacterial pathogens.

## 2 Materials and methods

### 2.1 Sample collection

Fresh leaves of *E*. *globulus* were collated from Islamabad, Pakistan. The plant was identified by Dr. Hassan Javed (Department of Plant Science) at Quaid I Azam University (QAU), Islamabad, and given a voucher specimen NCBI:txid34317. The sample was washed with DdH_2_O to eradicate the contaminations, then the fresh and washed leaves were cut into pieces (Chahomchuen et al., 2020).

### 2.2 Isolation of essential oils

To prepare leaves solution for oil extraction, 50 g fresh leaves were thoroughly mixed with 100 mL DdH_2_O in a 300 mL volumetric flask. Then the prepared solution was hydro-distilled for 3 h in triplicate using a Clevenger-type device, following the procedure prescribed in the European Pharmacopoeia (Pharmacopoeia (2005). To prepare for further analysis, the oils were dried up over anhydrous sodium sulfate, separated, and stored at −4°C. The volume of basic oil divided by the weight of the leaves (v/w) was used to calculate the percentage of fundamental oil substance present in the leaves.

### 2.3 Chemical analysis of essential oils and gas chromatography–mass spectrometry (GC–MS)

The chemical analysis and identification of bioactive compounds present in crude essential of *E. globulus* was done by using GCMS-QP 2010 Plus, Shimadzu, Japan working with electron ionization mode at 70 eV. Mass units were checked from 35 to 500 AMU. A DB-5 MS (30 m 0.25 mm id, 0.25 mm film thickness) fine segment. GC examination conditions and injector and indicator temperatures were the same as they were in this phase of the experiment as well (Stein et al., 2001), i.e., The oven’s temperature was set to rise from 50°C to 150°C at a rate of 3°C per minute, hold isothermal for 10 min, and then rise to 300°C at a rate of 10°C per minute. Peak area normalization was used to express the relative percentage of the chemical components in crude extracts as a percentage.

### 2.4 Collection of bacterial strains

The preserved standard bacterial cultures of *Cronobacter sakazakii (ATCC#29544), Pseudomonas aeruginosa (ATCC#10145), Listeria monocytogenes (ATCC#13932), Bordetella bronchiseptica (ATCC#4617,) Staphylococcus epidermidis (ATCC# 12228),* and *S. aureus (ATTC#9144),* were collected from pathology laboratory of a Biogen life sciences pharma Islamabad, Pakistan. The bacterial strains used were freeze-dried on Petri dish plates.

#### 2.4.1 Media preparation for juvenile culture

A nutritional media for juvenile culture was prepared by dissolving 6 g of nutrient broth in 100 mL of distilled water for the development of bacterial inoculation. When the pH was adjusted to 7.0, then autoclaved, and then put onto Petri plates (20 mL) in the laminar flow cabinet, the solution was ready for use.

#### 2.4.2 Strain isolation

A laminar flow cabinet was used to disinfect and label Petri dishes before *C*.*r sakazakii (ATCC#29544),* was introduced into one of the plates. All of the same procedures were used for the five bacteria, *P. aeruginosa (ATCC#10145), L. monocytogenes (ATCC#13932), B. bronchiseptica (ATCC#4617,) S. epidermidis (ATCC# 12228)* and *S*. *aureus (ATTC#9144)*. It was then incubated at 37°C for 24 h on all Petri plates. On Petri plates, the bacteria were now active and ready to multiply.

### 2.5 Assessment of antibacterial activities of essential oils

#### 2.5.1 Tested microorganisms (ATCC cultures)

For evaluating the *in-vitro* antimicrobial activity of the essential oils, a range of pathogenic Gram-negative and Gram-positive bacterial strains were selected from the American Type Culture Collection (ATCC). To initiate the experiment, all microbial cultures were streaked on agar slants and incubated at 3°C for 24 h. Subsequently, they were transferred to the broth for overnight growth.

### 2.6 Antibacterial activity of essential oils

#### 2.6.1 Procedure

A nutrient agar solution containing 6.3 g was dissolved in 120 mL of distilled water (DW). Subsequently, the agar solution and sterile Petri dishes underwent autoclaving at 121°C for 15 min. Post-sterilization, 40 mL of the agar solution was dispensed into each Petri dish and allowed to solidify for 20 min. Following solidification, the intended bacterial strain was inoculated onto each Petri dish utilizing the streak plate technique. After streaking, two wells were generated using a Cork borer and systematically labeled. Subsequently, 30 µL of Erythromycin (1 mg/mL) (used as the positive control) and 30 µL of the plant oil extract solution (1 mg/mL) were introduced into the respective wells. The Petri dishes were then incubated at 37°C for 24 h. The entire procedural operation occurred within a laminar flow cabinet under aseptic laboratory conditions (Magaldi et al., 2004).

### 2.7 Determination of minimum inhibitory concentration and minimum bactericidal concentration (MIC and MBC)

The assessment of MICs and MBCs for *E. globulus* Essential Oils (EOs) were carried out employing the microdilution technique within 96-well microplates (Newman and Cragg, 2007). The experimental procedures were conducted employing Mueller-Hinton (MH) broth as the growth medium. Microbial cell suspensions were rendered at a concentration of 10^6 colony-forming units per milliliter (cfu/mL) by three-fold dilution with 0.5% sodium chloride solution (0.5% NaCl). This dilution process achieved an optical density of 0.1 at 600 nm, as determined by a NanoDrop 2000c Spectrophotometer (Thermo Scientific, United States). Subsequently, the bacterial solution (100 µL) was added to each well of a 96-well plate.

Employing two-fold serial dilutions, a sequence of oil concentrations spanning from 4% to 0.625% was introduced to the subsequent wells, resulting in a final well volume of 100 µL. The negative control wells received 100 µL of MH broth, while the positive control wells contained 100 µL of bacterial inoculum devoid of the oil. The microplates were then incubated at 37°C for 24 h. After incubation, the optical density (OD) was quantified at 595 nm utilizing a microplate reader (iMark Microplate Reader, Bio-RAD, United States).

The determination of MIC was predicated on the lowest dilution displaying observable growth. In each well, 5 µL of a resazurin indicator solution was introduced. After a 30-min incubation at 37°C, the MIC assay outcomes were corroborated through color alteration. The presence of viable bacteria induced a discernible pinkish hue in the resazurin indicator. The MBC, signifying the concentration at which microbial growth was entirely inhibited and color alteration halted, was determined. The MBC was substantiated by applying 5 µL from the well corresponding to the MIC or higher concentrations onto MH agar plates. Following a 24-h incubation at 37°C, the absence of any growth on the agar corroborated the MBC value.

### 2.8 Time–kill studies

By measuring the percentage decrease in bacterial count over some time while in the presence of extract at its MIC, time–kill tests may be used to assess the antibacterial activity of EOs. The effectiveness of EOs against strains was determined by quantifying the decrease in CFU per milliliter during 24 h, as reported by Song et al. (2006). Quantification using this technique should not be more than 102 CFU.

### 2.9 Antioxidant activity

#### 2.9.1 DPPH assay

Measuring the ability of essential oils to search 2,20 -diphenyl-1-picryl hydrazyl (DPPH) stable fundamental was the key to determining their activity as an antioxidant. The assay was carried out spectrophotometrically according to the instructions provided by Harkat-Madouri et al. (2015); Chahomchuen et al. (2020), Shimada et al. (1992); Akolade et al. (2012). Ascorbic acid was used as standard solution and RSA (%) (µL/L AAE) was calculated the following formula.

$$\text{RSA }\left(\%\right)\;\left(µL/L\text{ AAE}\right)=\text{Abs STD }–\text{ Abs SPL}/\text{ Abs STD }\times 100$$

Where Abs STD is absorbance of standard and Abs SPL is absorbance of sample.

#### 2.9.2 Reduction ability by Fe^3+^-Fe^2+^Transformation

The method of EFabiyi et al. (2020) followed for the reduction ability of crucial oils were accessed using this process. Samples were compered with ascorbic standared caruve to find out AAE of the extracted oil using the following formula.

$$X=\left(y/0.0057\right),R^{2}=0.9647$$

### 2.10 Molecular docking methodology

The molecular docking methodology used in this study involved several software and tools. First, the crystal structures of the antioxidant and antimicrobial target proteins were obtained from the RCSB protein data bank. The PDB IDs of the proteins were 1AJ6 (National Center for Biotechnology Information, 1998) and 1R4U (RCSB, 2004). The decision to utilize the crystal structures of proteins (PDB IDs: 1AJ6 and 1R4U) for molecular docking in the study is considered due to their availability, potential functional relevance, and diversity of target representation. While the exact roles of these proteins in the metabolism of the selected bacteria were not explicitly mentioned, their selection is stem from factors such as known biological functions, structural characteristics, possible implications for human health, and the potential for uncovering novel interactions. These proteins also share binding site similarities with metabolic enzymes, facilitating insights into broader cellular mechanisms. Technical feasibility and compatibility with available software tools have further influenced their choice of docking targets. To prepare the protein structures for docking, we used BIOVIA’s Discovery Studio Visualizer (Morris et al., 2009) and Autodock tools (Visualizer, 2005; Bilal et al., 2022). This involved removing heteroatoms, co-crystal ligands, and solvent molecules, and optimizing the protein for docking. Next, the ligands were designed and drawn in ChemDraw Ultra (Channar et al., 2022), and their energy was minimized using Chem3D Pro (Ferreira et al., 2015). The ligands were then converted to pdbqt files using OpenBabel GUI (Zentgraf et al., 2007). In AutoDock, the grid box was built using a size 60 × 60 × 60 Å, pointing for 1AJ6 and 1R4U receptors, X = 60.02, Y = −7.77, Z = 34.74 and X = 44.35, Y = 40.91, Z = 30.47 coordinates, respectively, with a grid point spacing of 0.375 Å for each. The centre of the grid box was adjusted to cover the active pocket. To perform the docking, we used Autodock vina (Yusuf et al., 2008; Morris et al., 2009; Trott and Olson, 2010; Eberhardt et al., 2021). The ligands were docked into the active site of the protein, and the resulting ligand-protein interactions were analyzed using BIOVIA’s Discovery Studio Visualizer.

### 2.11 Statistical analysis

With the help of Microsoft Excel 2013, the mean value and standard deviations were determined. Analysis of variance (Ahmadov et al., 2020) was used to analyze the data by determining the differences between means for implication at P 0.05 using SPSS’s Duncan’s numerous range test (version 16.0) (Khan et al., 2019).

## 3 Results and discussion

### 3.1 Isolation and identification of *E*. *globulus*

Fresh leaves of *E*. *globulus* were collated from Islamabad. The plant was identified with the help of a taxonomist at QAU, Islamabad. The samples were washed with DdH_2_O to eradicate the contamination, and then the fresh and washed leaves were cut into pieces.

### 3.2 Isolation of essential oils

**FIGURE 1:**
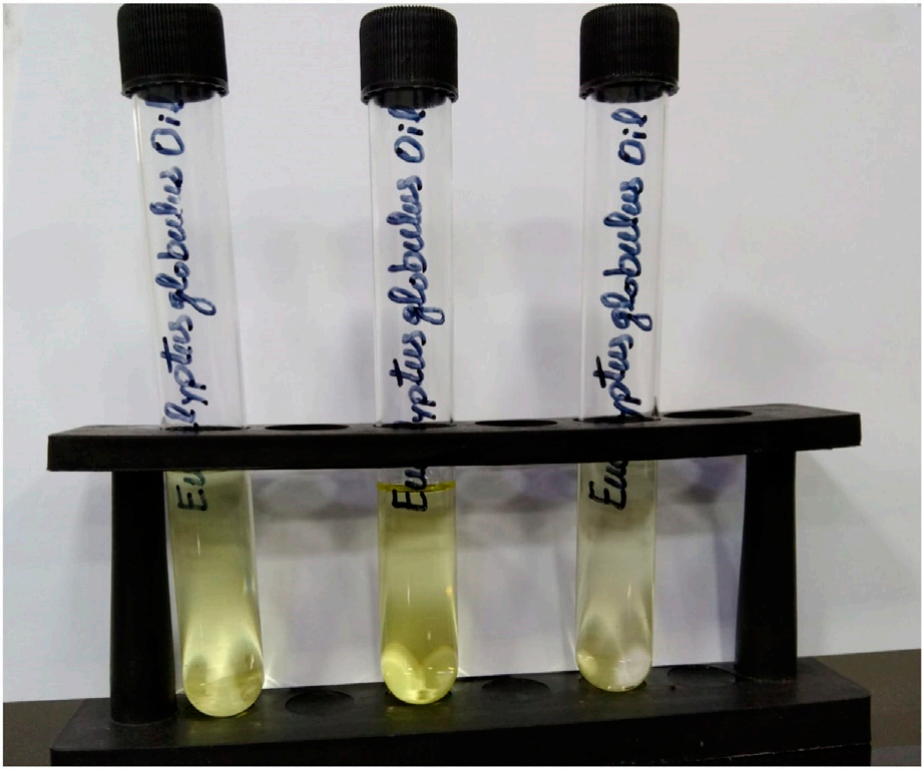
Extracted oil from *E*. *globulus*.

Essential oils were by using a Clevenger-type device, following the procedure prescribed in the European Pharmacopoeia, according to the protocol (Yadav et al., 2022). To prepare for further analysis, the oils were dried up over anhydrous sodium sulfate, separated, and stored at - 4°C. The volume of basic oil divided by the weight of the leaves (v/w) was used to calculate the percentage of fundamental oil substance present in the leavesi.e., 1 mL/100 g leaves (Figure 1).

### 3.3 Detection of the bioactive compound using GC-MS

**FIGURE 2:**
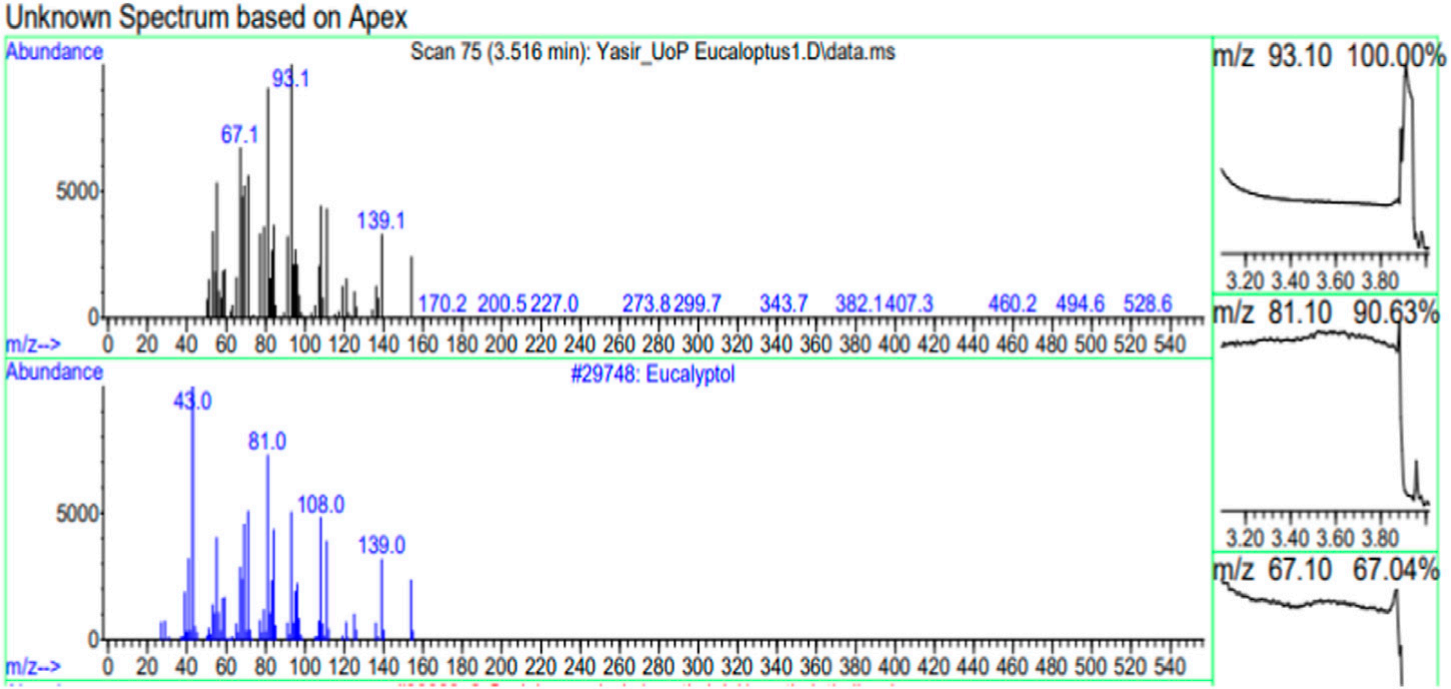
GC-MS chromatogram of Eucalyptol base peak (m/z) 93.10.

**FIGURE 3:**
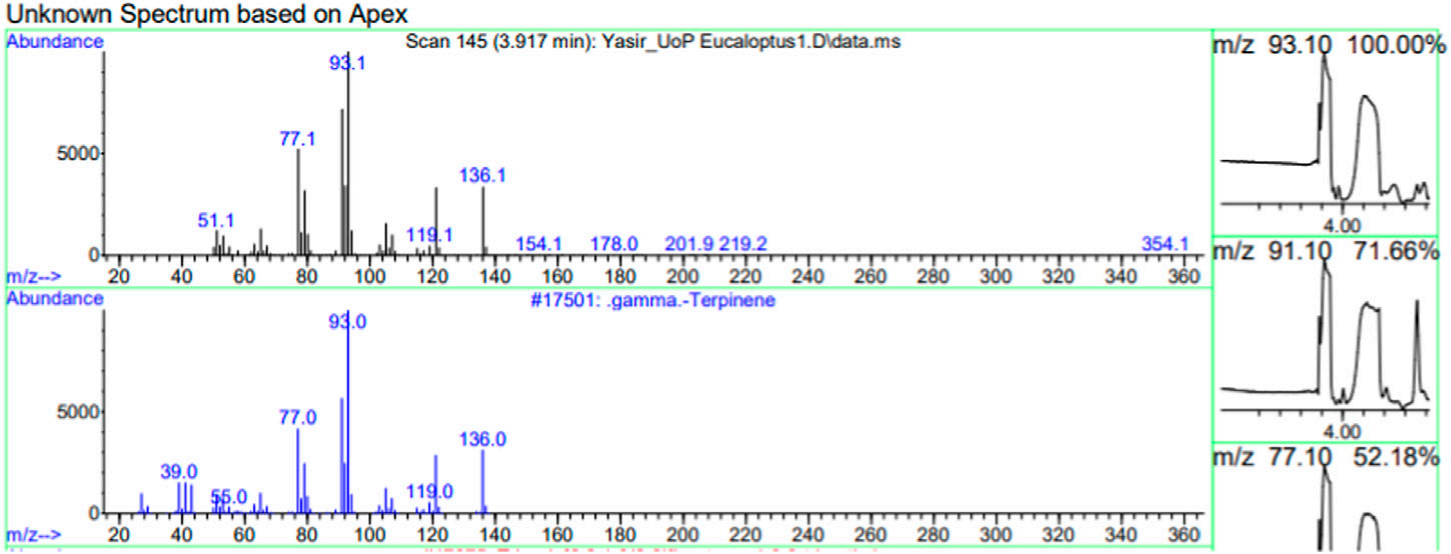
GC-MS chromatogram of Gama-Terpinene base peak (m/z) 93.10.

**FIGURE 4:**
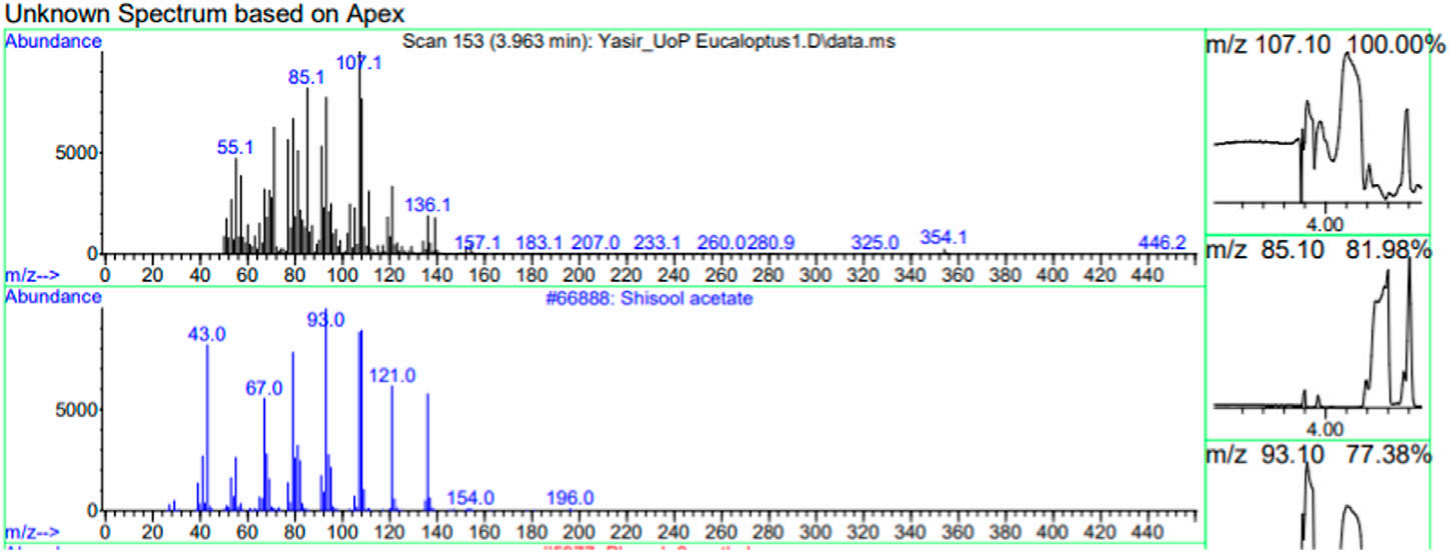
GC-MS chromatogram of Shisool acetate base peak (m/z) 107.10.

**FIGURE 5:**
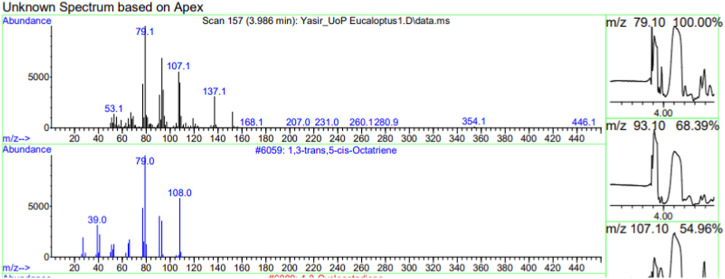
GC-MS chromatogram of 1,3-trans,5-is-Octatriene base peak (m/z) 79.10.

**FIGURE 6:**
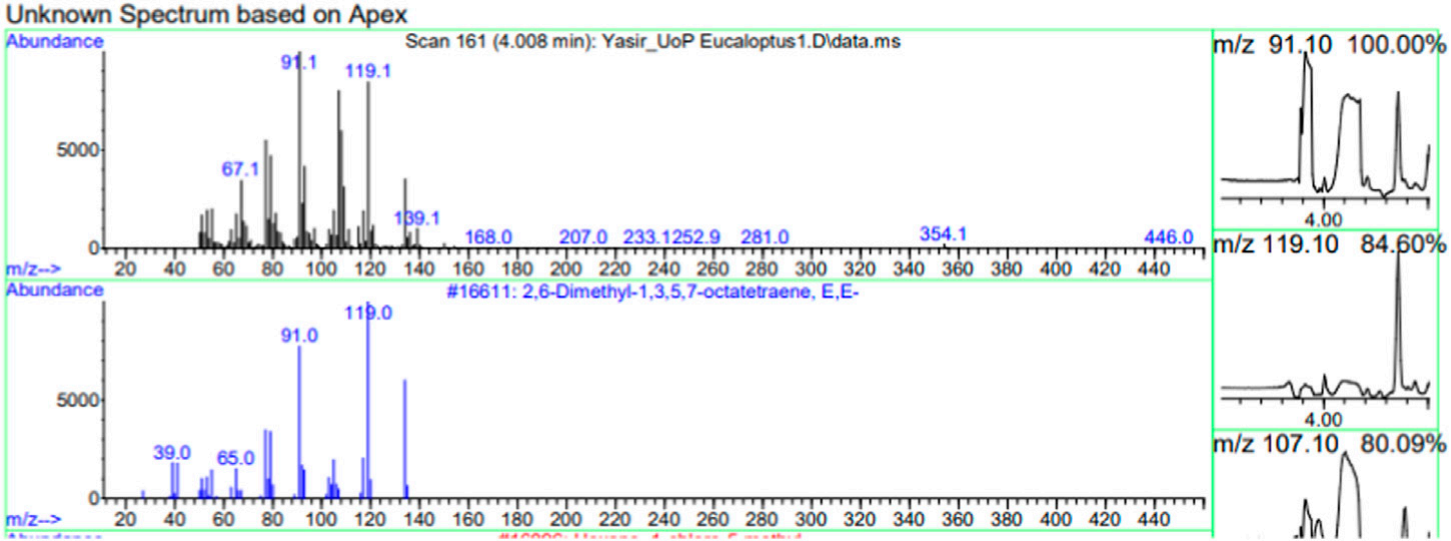
GC-MS chromatogram of 2,6-Dimethyl-1,3,5,7-octatetraene, E,E base peak (m/z) 91.10.

**FIGURE 7:**
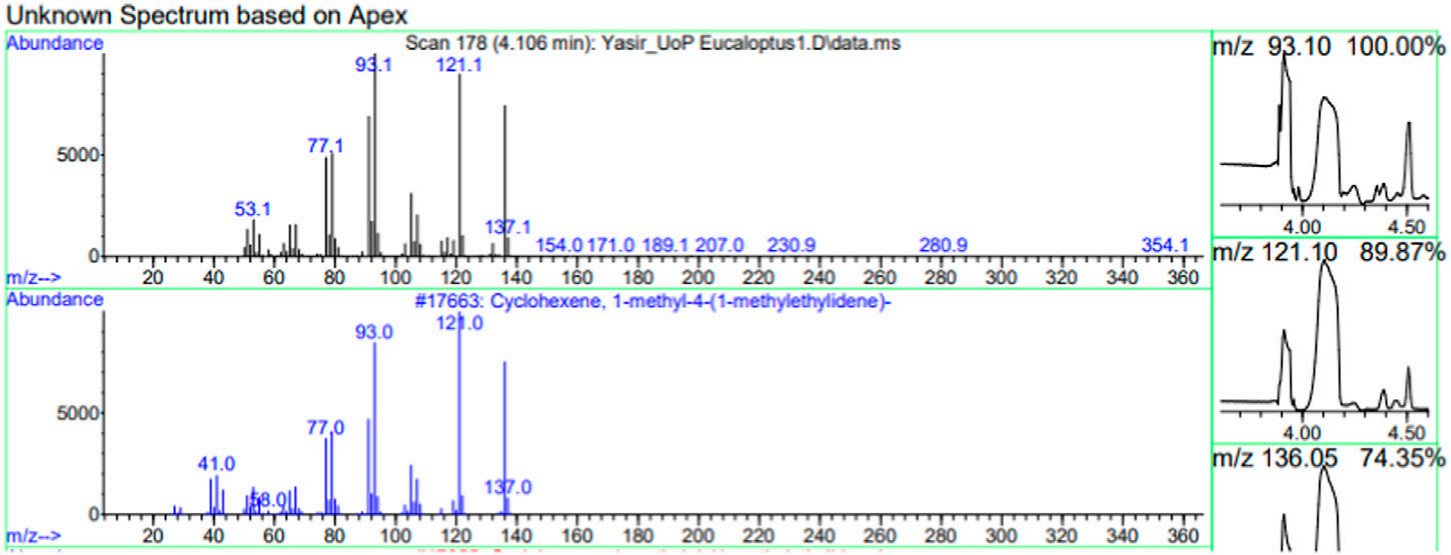
GC-MS chromatogram Cyclohexene, 1-methyl-4-(1-methylethylidene) base peak (m/z) 91.10.

The constituents of the yellow volatile oil of *E*. *globulus* were detected by GC-MS Chromatogram, and the compounds in the oil extract were characterized based on GC-MS evidence with different retention times (Supplementary Figure S1). The analysis of complex mixtures such as crude oil identified some low molecular weight compounds Eucalyptol, Gamma.-Terpinene, Shisool acetate, 1,3-trans,5-cis-Octatriene, 2,6-Dimethyl-1,3,5,7-octatetraene, E, E, Cyclohexene, 1-methyl-4-(1-methylethylidene)-, Benzene, 1-methyl-4-(1-methylethenyl)- Butanoic acid, 3-methyl-, 3-methylbutyl ester, 1,3,8-p-Menthatriene shown in (Table 1). In addition to that several other compounds were also found, i.e., Fenchol 2-Methyl-trans-3a,4,7,7a-tetrahydroindane, (E,E,E)-2,4,6-Octatriene, 1,2,3,6-Tetrahydrobenzylalcohol, acetate, Alloaromadendrene,Phenol, 2-ethyl-4,5-dimethy,Phenol, 2-methyl-5-(1-methylethyl)-,p-Cymen-7-ol,1,5,5-Trimethyl-6-methylene-cyclohexene, 1,3-Cyclohexadiene, 1-methyl-4-(1-methylethyl)-,2,6-Octadien-1-ol, 3,7-dimethyl-, acetate, (Z) *etc.* The large compound fragments compared to small compounds gave a taller appearance of peaks at different m/z ratios for 28 min (Figures 2–7). The small peaks could be caused by bioactive compounds that are present in small amounts or by major compounds that have broken down. Due to these bio-active components, in the last several years, the application of Eucalyptus oil has become a significant field of health- and medical-related study. Earlier research was also documented by Sharma, and Kaur, (Pharmacopoeia, 2005).

**TABLE 1:** List of essential compounds in E. globulus oil extract identified by using GC-MS.

S.No	Compound name	Retention time RT (Min)	Mass/Charge m/z
1	Eucalyptol	3.516	93.10
2	γ -Terpinene	3.917	93.10
3	Shisool acetate	(3.963	107.10
4	1,3-trans,5-cis-Octatriene	3.986	79.10
5	2,6-Dimethyl-1,3,5,7-octatetraene, E,E	4.008	91.10
6	Cyclohexene, 1-methyl-4-(1-methylethylidene)-	4.106	93.10
7	Benzene, 1-methyl-4-(1-methylethenyl)-	4.174	117.10
8	Butanoic acid, 3-methyl-, 3-methylbutyl ester	4.255	70.10
9	1,3,8-p-Menthatriene	4.358	91.10
10	Fenchol	4.398	85.10
11	2-Methyl-trans-3a,4,7,7a-tetrahydroindane	4.449	79.10
12	(E,E,E)-2,4,6-Octatriene	4.512	108.10
13	1,2,3,6-Tetrahydrobenzylalcohol, acetate	4.581	79.10
14	Alloaromadendrene	9.078	91.10
15	Phenol, 2-ethyl-4,5-dimethy	7.007	135.10
16	Phenol, 2-methyl-5-(1-methylethyl)-	7.053	135.10
17	p-Cymen-7-ol	7.167	135.10
18	1,5,5-Trimethyl-6-methylene-cyclohexene	7.316	121.10
19	1,3-Cyclohexadiene, 1-methyl-4-(1-methylethyl)-	7.482	121.10
20	2,6-Octadien-1-ol, 3,7-dimethyl-, acetate, (Z)	7.739	69.10

According to *Sumitha & Thoppil*, (2016), analyzing the chemical composition of leaf essential oil was accomplished using gas chromatography-mass spectrometry (GC/MS). GC/MS research revealed that *O. gratissimum* essential oil consists of 51 distinct components. 4-(5-methyl-1-azabicyclo (2, 2) octan-2), and 1-(2, 5-dimethoxyphenyl)-propyl-benzene were the most common compounds (5.53 percent). 24 h after inhaling the essential oil, the oil had an LC_50_ value of 26.10 parts per million, and an LC_90_ value of 82.83% of that value (Stein et al., 2001).

In another study, *Panyajai, et al., 2020* studied that The Algerian wild plant *Elaeoselinum thapsioides* had its leaves and stem hydro-distilled to get an essential oil. This oil was analyzed by GC-MS for the first time. 45 compounds were found, which made up 93.8 percent of the total oil. The oil had a high amount of hydrocarbon derivatives of monoterpenes (75.9 percent). Myrcene made up 61% of the essential oil, followed by germacrene D (10.3%), -pinene (6.5%), and -pinene (2.9%).

### 3.4 Assessment of the antibacterial activities of crucial oils

#### 3.4.1 Antibacterial activity

**FIGURE 8:**
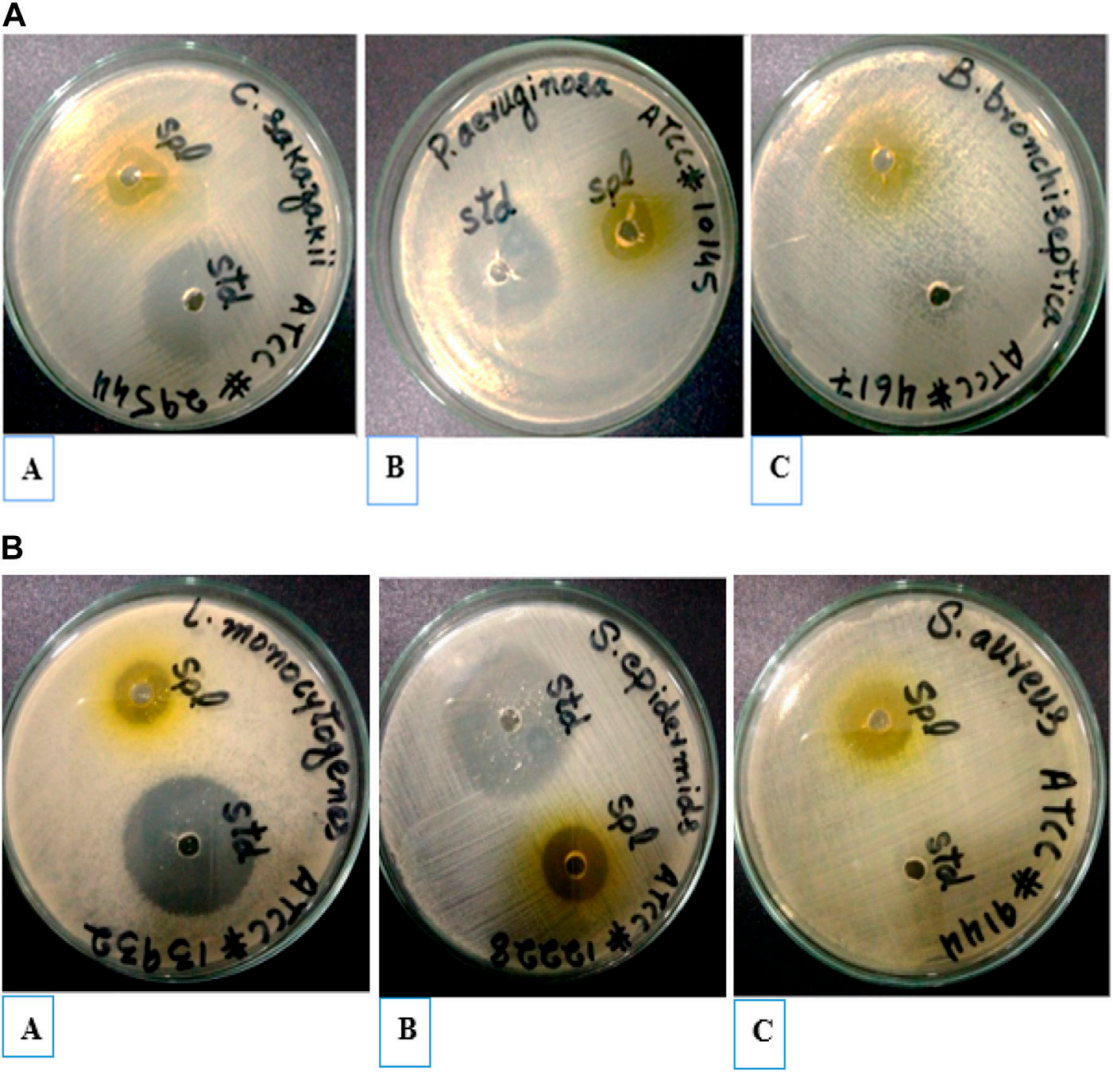
**(A)** Inhibition zone of extracted oil of *E*. *globulus* against Gram-negative (Std) positive control (Spl). **(B)** Inhibition zone of extracted oil of *E*. *globulus* against Gram-positive (Std) positive control (Spl).

**FIGURE 9:**
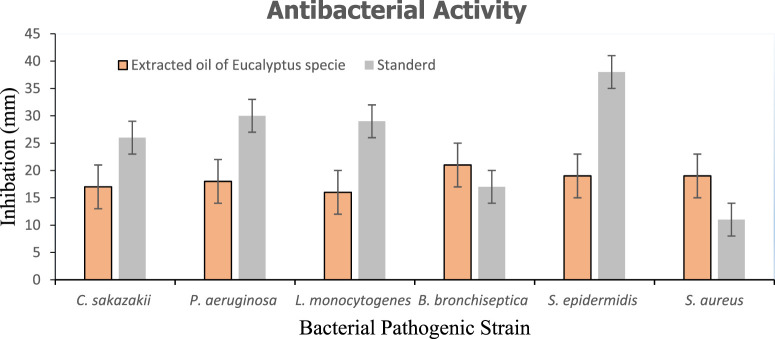
Inhibition zone of extracted oil of *E*. *globulus* against Gram-negative and Gram-positive.

Antibacterial activities of extracted oil of *E*. *globulus* were investigated against various pathogenic bacteria, such as *C. sakazakii (ATCC#29544), P. aeruginosa (ATCC#10145), L. monocytogenes (ATCC#13932), B. bronchiseptica (ATCC#4617) S. epidermidis (ATCC# 12228)* and *S. aureus (ATTC#9144)* using the well diffusion method. Extracted oil of *E*. *globulus* showed antibacterial activity against both Gram-positive and negative bacteria. The diameter of inhibition zones (mm) around each well with extracted oil of *E*. *globulus* is represented in (Figures 8A, B). The antimicrobial activity of extracted oil of *E. globulus* was found against (Figure 9A) (A) (*Cronobactersakazakii* (Zone of inhibition 17 mm), (B) *P*. *aeruginosa* (18 mm), (C) *L*. *monocytogenes* (Zone of inhibition 16 mm), (Figure 9B), (A) *B*. *bronchiseptica* (21 mm), (B) *S*. *epidermidis* (Zone of inhibition 19 mm), and (C) *S*. *aureus* (Zone of inhibition 19 mm), pathogenic bacterial strains.

Many researchers have investigated the antibacterial properties of essential oils and different polarity of crude plant extracts against a variety of bacterial strains. (Magaldi et al., 2004; Song et al., 2006). They found that the essential oils had a strong antibacterial effect on the bacterial strains they were tested against. The primary chemical components are responsible for the plant essential oil’s considerable biological actions. This research work shows great antibacterial activities against both Gram-negative and Gram positive similar to as reported in previous literature.

### 3.5 Time–kill studies

Bacterial growth was measured when exposed to Eos at the minimum inhibitory concentration (MIC) (Figure 9). In fact, after just 1 h of incubation, bacterial growth was decreased when Eos was present at concentrations comparable to their MIC. This significant impact may be attributable to carvacrol’s ability to depolarize and weaken the membrane that surrounds the cytoplasm. These tissues are a vital biological target for EO components because of their hydrophobic nature. Some antimicrobials, like thyme, can kill microbes by destroying their whole cell membranes. Also, carvacrol and thymol are structural isomers in that they vary in the position of a hydroxyl group on the phenolic ring. The hydroxyl group makes them more hydrophilic, which may facilitate their solubility in the microbial membrane and subsequent alterations to the membrane.

### 3.6 Effects of *E.s globulus* oil on antibacterial activity, MIC and MBC

MICs and MBCs were determined for the five reference strains listed in (Table 2) to assess the antibacterial activity of the *E*. *globulus* oil extract. The minimum inhibitory concentration (MIC) for *C*. *sakazakii (ATCC#29544),* was 0.4% (v/v), whereas the minimum inhibitory concentration (MBC) was 3% (v/v) (Table 2). The minimal inhibitory concentration (MIC) and minimum inhibitory concentration (MBC) values for *P. aeruginosa* (*ATCC#10145, L. monocytogenes (ATCC#13932), B. bronchiseptica (ATCC#4617) S.s epidermidis (ATCC# 12228)* and *S. aureus (ATTC#9144)* were all 2% (v/v) and 4% (v/v), respectively. These findings suggested that oil extracted from *E. citriodora* might be antibacterial against the microorganisms used in the study. This was discovered by examining the cytotoxic and antibacterial effects on fibroblast cell lines (MIC, MBC and agar disc diffusion technique). As with NLP-subcytotoxic ZMEO, the results were better in comparison to MIC/MBC values for multidrug-resistant bacteria and the chlorhexidine dosages examined (Shimada et al., 1992). Our result also was comparable to the above literature.

**TABLE 2:** Minimal inhibitory concentrations (MIC) and minimum bactericidal concentrations (MBC) for the antibacterial activity of *E*. *globulus* oil.

Bacterial strains	MIC(μg/mL)	MBC(μg/mL)
*Cronobacter sakazakii*	0.412	3.02
*Pseudomonas aeruginosa*	2.23	4 .21
*Listeria monocytogenes*	2.03	4.04
*Bordetella bronchiseptica*	2.15	4.07
*Staphylococcus epidermidis*	2.04	4.34
*Staphylococcus aureus*	2.07	4.26

### 3.7 Free radical scavenging activity of extracts by DPPH

**FIGURE 10:**
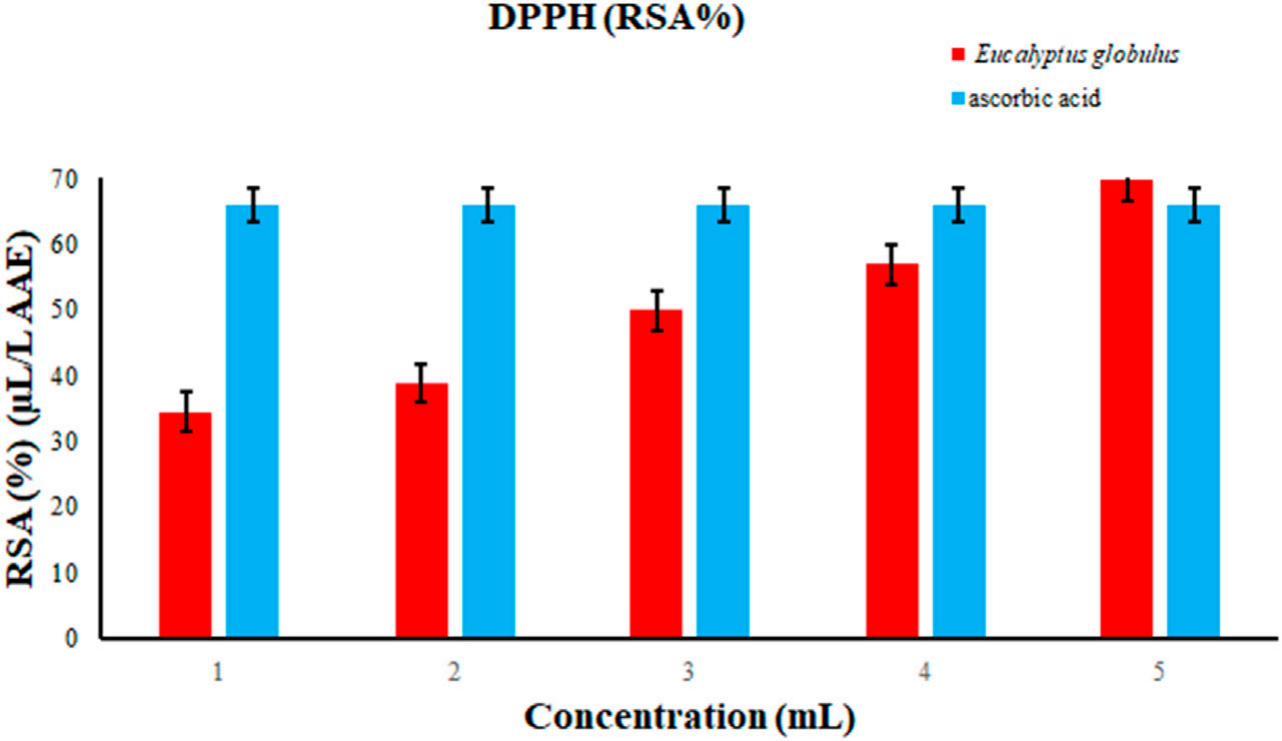
DPPH RSA (%) (µL/L AAE) of essential oil extracted from *E. globulus*.

In the present study antioxidant activities of essential oil extracted from *Eucalyptus globulus* were estimated by DPPH. Polyphenols have an ability that is called as H-ion donating ability due to which a toxic and reactive oxygen species could be converted to nontoxic one. For determining the antioxidant activity, the reaction with DPPH was hoped to be quick in action due to its rapid switching of labile hydrogen to radicals. There was formation of purple colored solution and absorbance was taken at 517 nm. Standard of a curve of Ascorbic acid. Antioxidants were present in the Eucalyptus extracts that faded the dark colour of the solution. Technique used for extraction was maceration and DPPH assay final calculations were shown as ascorbic acid equivalent of Eucalyptus extracts. Methanolic extract was used for testing Eucalyptus is scavenging activity (Figure 10).

### 3.8 Ferric reducing antioxidant power assay (FRAP) assays

**FIGURE 11:**
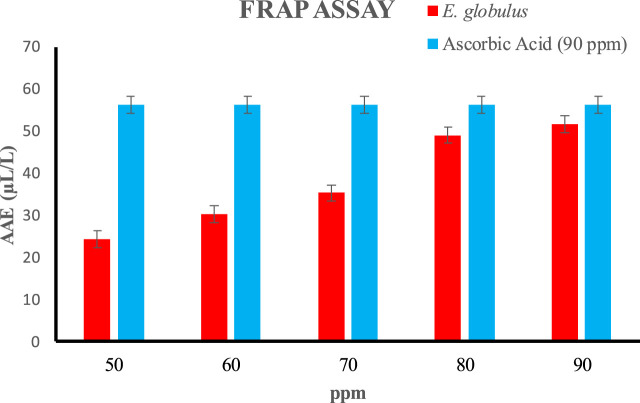
FRAP assay of essential oil extracted from *E*. *globulus* (AAE (µL/L).

The FRAP method is the sharp, less difficult, cheap, renewable, and highly achievable method. Antioxidant contents have a significantly high reducing power due to which they can easily and effectively reduce ferric to ferrous ions. As the concentration of extract is increased this reducing power will also increase. In the present research work,a regular increase in the reduction potential was observed as the concentration of the tested extract increased. The reduction potential of the oil extracted from *E*. *globulus* was measured over the concentrations ranging from 50 to 90 ppm showing 24.26 to 51.56 AAE (µL/L) shown in (Figure 11). Antioxidants may reduce compounds because they may stabilize radicals by giving up electrons or hydrogen atoms to make radicals less reactive. Osawa et al. reported that *E. cinerea, E. cosmophylla, E. globulus, E. perriniana* and *E. viminalis* have antioxidant properties due to the presence of 𝛽-diketone and ellagic acid. In another study, Singhat al., 2012 analysis of essential oil by gaschromatography–mass spectrometry (GC–MS) revealed the presence of 43 components constituting 99.2% of the oil of *E. citriodora*. This research work also shows antioxidant activities similar to previous literature.

*Eucalyptus globulus* oil is utilized in pharmaceutical preparations as an anti-diabetic, antioxidative, antiviral, antibacterial, antifungal, antitumor, antihistaminic, anticancer cytochrome p450 inhibitor and hepatoprotective agent, as well as in goods like cough sweets, lozenges, ointments, toothpaste, and inhalants. Extracted oil of *E*. *globulus*, its potential health benefits because safe and effective, extracted oil of *E*. *globulus* product can be widely used as a medicinal plant using GC-MS technique as a rapid, simple, low cost for low molecular weight organic analysis.

### 3.9 Molecular docking

Table 3 provides information on the ligands that bind to the antioxidant protein with the code 1AJ6, along with their binding scores (in Kcal/mol) and the residues in the binding pocket of the protein that interact with the ligands, along with the distance (Fabiyi et al., 2020) between the ligand and the interacting residues. The table lists 18 ligands, numbered from 1 to 18. For each ligand, the chemical name, binding score, and interacting residues with their distances are listed. The interacting residues are listed using their three-letter amino acid code followed by the position number in the protein sequence in parentheses. The distance between the ligand and the residue is given in angstroms (Å).

**TABLE 3:** Ligand Codes with Chemical names, Molecular docking scores and binding interactions with distance for 1AJ6 and 1R4U.

		1AJ6 (antioxidant protein)		1R4U (anti-microbial protein)	
Ligand Code	Chemical Name	Binding Score Kcal/mol	Binding Pocket Interaction Residues and Distance Å	Binding Score Kcal/mol	Binding Pocket Interaction Residues and Distance Å
1	Eucalyptol	−4.8	GLY119 (3.45)	−5.1	AGR105 (2.74)
			IIE94 (5.26)		TRP208 (3.91)
			ALA100 (4.30)		AGR105 (5.05)
					AGR128 (5.26)
2	Gamma. -Terpinene	−5.6	Asn46(3.55)	−5.0	
			Glu50 (4.56)		
			Asp73(5.23)		CYS103(3.90)
			Ile78 (2.33)		PRO76 (4.38)
			Met95 (3.45)		CYS103(4.47)
			Val120(4.56)		TYR30 (4.86)
			Thr165 (2.94)		PRO76 (5.38)
			Val167(3.67)		ARG128 (5.33)
3	1,3-trans,5-cis-Octatriene	−4.1	VAL43(5.16)	−4.3	VAL73(5.28)
			MET95 (4.98)		PRO76 (5.01)
			ALA47 (4.04)		PRO76 (4.26)
			VAL71(5.07)		CYS103 (4.05)
			VAl167(4.16)		CYS103 (4.22)
			VAL120(4.61)		MET32 (4.72)
			ILE78 (4.28)		MET32 (3.59)
					CYS103(3.83)
					TYR30 (4.86)
4	2,6-Dimethyl-1,3,5,7-octatetraene, E,E	−4.2	VAL43(5.30)	−4.8	
			ALA47 (4.46)		
			VAL120(4.00)		CYS103 (3.77)
			VAL167(5.22)		TYR30 (5.09)
			VAL167(4.91)		TYR30 (5.17)
			ILE78 (4.88)		
5	Cyclohexene, 1-methyl-4-(1-methylethylidene)	−6.0	ILE78 (5.07)	−5.4	CYS103 (5.10)
			VAL43(4.43)		TYR30 (4.83)
			VAL120(3.99)		
			VAL167(4.41)		
			GLU50 (2.39)		
			THR165 (3.93)		
			ASN46(2.99)		
6	Benzene, 1-methyl-4-(1-methylethenyl)	−5.5		−5.0	TRP208 (5.24)
					TRP208 (4.02)
					ARG105 (4.52)
			ASN46(3.66)		ARG128 (4.28)
			VAL43(4.33)		CYS103 (4.67)
			VAL120(3.65)		ARG105 (3.98)
			VA:167 (4.35)		ARG128 (4.35)
			ILE78 (4.94)		TRP208 (5.07)
			THR165 (4.39)		TRP208 (4.72)
			MET95 (5.90)		TRP208 (4.91)
			GLU50 (3.98)		ARG128 (5.41)
7	Butanoic acid, 3-methyl-, 3-methylbutyl ester	−5.8	THR165 (2.79)	−4.9	TRP106 (1.95)
			ILE78 (5.39)		THR107 (2.84)
			ILE78 (4.36)		CYS103(3.89)
			MET95 (4.48)		TYR30 (5.14)
			VAL120(3.90)		
			LEU132 (5.46)		
			VAL167(4.77)		
8	1,3,8-p-Menthatriene	−5.5	ASN46(3.73)	−5.0	ARG105 (4.78)
			VAL43(4.47)		ARG128 (4.22)
			VAL120(3.68)		CYS103(4.65)
			VAL167(4.47)		TRP208 (4.87)
			ILE78 (4.31)		TRP208 (4.78)
			MET95 (5.00)		ARG105 (3.88)
			VAL120(4.01)		ARG128 (4.94)
			VAL167(4.80)		
			ILE78 (4.88)		
9	Fenchol	−4.5	ILE94 (5.41)	−5.2	HIS256(2.17)
			ALA100 (3.91)		ARG176 (5.15)
10	(E,E,E)-2,4,6-Octatriene	−4.5	ALA47 (4.23)	−3.8	CYS103(3.89)
			ALA47 (3.80)		ARG105 (4.02)
			ILE78 (5.27)		MET32 (4.14)
			ILE78 (4.96)		VAL73(4.82)
			VAL43(4.87)		CYS103(4.16)
			VAL71(4.32)		TYR30 (5.06)
			VAL167(4.97)		TYR30 (5.09)
11	1,2,3,6-Tetrahydrobenzylalcohol, acetate	−5.8	GLY77 (2.10)	−5.3	TRP106 (1.96)
			THR165 (2.21)		MET32 (5.42)
			ALA47 (5.30)		PRO76 (5.20)
					CYS103 (3.79)
					TYR30 (4.91)
12	Alloaromadendrene	−6.2	Arg190 (4.37)	−6.9	TRP208 (3.84)
			PHE41(5.06)		CYS103 (4.35)
			PHE41(4.14)		CYS103(3.74)
					TYR30 (5.24)
13	Phenol, 2-ethyl-4,5-dimethyl	−5.7	ASP73(2.50)	−5.5	GLU31 (2.20)
			THR165 (3.79)		TYR30 (4.86)
			ALA47 (4.42)		PRO76 (3.99)
			VAL43(4.81)		CYS103(4.17)
			VAL71(4.42)		TYR30 (5.31)
			VAL167(4.43)		TYR30 (5.34)
			ILE78 (5.34)		CYS103(4.57)
14	Phenol, 2-methyl-5-(1-methylethyl)	−6.1	ASN46(2.36)	−5.6	GLU31 (2.02)
			ASN46(3.60)		GLU31 (2.27)
			VAL43(4.30)		HIS104(2.70)
			VAL120(3.66)		CYS103(4.16)
			VAL167(4.43)		TYR30 (4.88)
			ILE78 (5.05)		
15	p-Cymen-7-ol	−5.7	VAL71(2.55)	−5.2	VAL29(3.09)
			THR165 (2.76)		VAL29(3.22)
			THR165 (3.61)		TRP106 (3.58)
			ALA47 (4.92)		TYR30 (4.80)
					CYS103(5.11)
16	1,5,5-Trimethyl-6-methylene-cyclohexene	−4.5	ILE94 (5.42)	−5.1	PRO76 (5.38)
			ALA100 (4.32)		CYS103(4.24)
					TYR30 (4.90)
17	1,3-Cyclohexadiene, 1-methyl-4-(1-methylethyl)	−5.6	ASN46(3.64)	−5.0	CYS103(3.94)
			VAL43(4.31)		PRO76 (4.33)
			VAL120(3.69)		CYS103(4.47)
			VAL167(4.44)		TYR30 (4.91)
			ILE78 (5.07)		PRO76 (5.38)
					ARG128 (5.28)
18	2,6-Octadien-1-ol, 3,7-dimethyl-, acetate, (Z)	−5.0	ARG76 (2.72)	−5.2	GLU31 (2.41)
			ILE78 (5.39)		ARG105 (3.92)
			VAL120(5.11)		ARG128 (4.21)
			ILE78 (5.39)		ARG105 (3.72)
			VAL43(4.34)		TRP208 (4.94)
			VAl120(3.76)		TRP208 (4.31)
			VAL167(4.45)		
Native Ligand	Novobiocin (1AJ6) and Oxonic Acid (1R4U)	−6.4	ARG76 (4.01)	−4.8	THR74 (3.35)
			GLU50 (3.48)		TYR30 (4.89)
			GLY77 (4.53)		CYS103 (4.14)
			ILE78 (5.11)		
			PRO79 (5.16)		

Upon analyzing the ligand binding scores and the interaction residues of the antioxidant protein 1AJ6, we can compare the different ligands’ binding affinities to the protein as shown in Table 3 and Supplementary Table S5. Eucalyptol has a binding score of −4.8 kcal/mol and interacts with GLY119, IIE94, and ALA100 residues. Gamma-Terpinene has the highest binding affinity with a score of −5.6 kcal/mol and interacts with Asn46, Glu50, Asp73, Ile78, Met95, Val120, and Thr165 residues. The 1,3-trans,5-cis-Octatriene has a binding score of −4.1 kcal/mol and interacts with VAL43, MET95, ALA47, VAL71, VAL167, VAL120, and ILE78 residues. 2,6-Dimethyl-1,3,5,7-octatetraene, E,E has a binding score of −4.2 kcal/mol and interacts with VAL43, ALA47, VAL120, VAL167, and ILE78 residues. Cyclohexene, 1-methyl-4-(1-methylethylidene)- has a binding score of −6.0 kcal/mol and interacts with ILE78, VAL43, VAL120, VAL167, GLU50, THR165, and ASN46 residues. Benzene, 1-methyl-4-(1-methylethenyl)- has a binding score of −5.5 kcal/mol and interacts with ASN46, VAL43, VAL120, VAL167, ILE78, THR165, MET95, and GLU50 residues. Butanoic acid, 3-methyl-, 3-methylbutyl ester has a binding score of −5.8 kcal/mol and interacts with THR165, ILE78, MET95, VAL120, LEU132, and VAL167 residues. 1,3,8-p-Menthatriene has a binding score of −5.5 kcal/mol and interacts with ASN46, VAL43, VAL120, VAL167, ILE78, and MET95 residues. Fenchol has a binding score of −4.5 kcal/mol and interacts with ILE94 and ALA100 residues (E,E,E)-2,4,6-Octatriene has a binding score of −4.5 kcal/mol and interacts with ALA47, ILE78, VAL43, VAL71, and VAL167 residues. 1,2,3,6-Tetrahydrobenzylalcohol, acetate has a binding score of −5.8 kcal/mol and interacts with GLY77, THR165, and ALA47 residues. Alloaromadendrene has the highest binding affinity with a score of −6.2 kcal/mol and interacts with Arg190 and PHE41 residues. Phenol, 2-ethyl-4,5-dimethy has a binding score of −5.7 kcal/mol and interacts with ASP73, THR165, ALA47, VAL43, VAL71, VAL167, and ILE78 residues. Phenol, 2-methyl-5-(1-methylethyl)- has a binding score of −6.1 kcal/mol and interacts with ASN46, VAL43, VAL120, VAL167, ILE78, and THR165 residues. p-Cymen-7-ol has a binding score of −5.7 kcal/mol and interacts with VAL71, THR165, and ALA47 residues. 1,5,5-Trimethyl-6-methylene-cyclohexene has a binding score of −4.5kval.mol, indicating a relatively strong binding. The amino acid residues involved in the interaction are ILE94 and ALA100, which are both hydrophobic amino acids, and the distance between the ligand and these residues is 5.42 and 4.32 Å, respectively. 1,3-Cyclohexadiene, 1-methyl-4-(1-methylethyl)-has a higher binding score of −5.6, indicating an even stronger binding affinity. The amino acid residues involved in the interaction are ASN46, VAL43, VAL120, VAL167, and ILE78, which are all hydrophobic residues, and the distances range from 3.64 to 5.07 Å. Lastly, 2,6-Octadien-1-ol, 3,7-dimethyl-, acetate, (Z), the binding score is −5.0, indicating strong binding. The amino acid residues involved in the interaction are ARG76, ILE78, VAL120, VAL43, and VAL167, which are also hydrophobic residues, and the distances range from 2.72 to 5.39 Å. Among the analyzed ligands, Alloaromadendrene showed the highest binding affinity of −6.9 kcal/mol, surpassing the native ligand novobiocin’s binding score of −6.4 kcal/mol against the antimicrobial protein 1R4U.

The results of the molecular docking analysis of 18 ligands against the anti-microbial protein 1R4U were obtained, and the binding scores and the interacting residues and distance in the binding pocket were recorded and presented in Table 3 and shown in Supplementary Table S6. The analysis revealed that the ligand Eucalyptol has the highest binding score of −5.1 kcal/mol, and it interacts with the residues AGR105, TRP208, AGR105, and AGR128, with distances of 2.74, 3.91, 5.05, and 5.26 Å, respectively. Gamma.-Terpinene had the second highest binding score of −5.0 kcal/mol, and it interacts with the residues CYS103, PRO76, CYS103, TYR30, PRO76, and ARG128, with distances of 3.90, 4.38, 4.47, 4.86, 5.38, and 5.33 Å, respectively. 1,3-trans,5-cis-Octatriene had a binding score of −4.3 kcal/mol and interacted with the residues VAL73, PRO76, PRO76, CYS103, CYS103, MET32, MET32, CYS103, and TYR30, with distances of 5.28, 5.01, 4.26, 4.05, 4.22, 4.72, 3.59, 3.83, and 4.86 Å, respectively. 2,6-Dimethyl-1,3,5,7-octatetraene, E,E had a binding score of −4.8 kcal/mol and interacted with the residues CYS103, TYR30, TYR30, with distances of 3.77, 5.09, and 5.17 Å, respectively. Cyclohexene, 1-methyl-4-(1-methylethylidene)- had a binding score of −5.4 kcal/mol and interacted with the residues CYS103 and TYR30, with distances of 5.10 Å and 4.83 Å, respectively. Benzene, 1-methyl-4-(1-methylethenyl)- had a binding score of −5.0 kcal/mol and interacted with the residues TRP208, TRP208, ARG105, ARG128, CYS103, ARG105, ARG128, TRP208, TRP208, TRP208, and ARG128, with distances of 5.24, 4.02, 4.52, 4.28, 4.67, 3.98, 4.35, 5.07, 4.72, 4.91, and 5.41 Å, respectively. Butanoic acid, 3-methyl-, 3-methylbutyl ester had a binding score of −4.9 kcal/mol and interacted with the residues TRP106, THR107, CYS103, and TYR30, with distances of 1.95, 2.84, 3.89, and 5.14 Å, respectively. 1,3,8-p-Menthatriene exhibited a binding affinity of −5.0 kcal/mol and was found to interact with key residues such as ARG105, ARG128, CYS103, and TRP208. Ligand 9, Fenchol, had a slightly higher binding affinity of −5.2 kcal/mol and interacted with HIS256 and ARG176. Ligand 10, (E,E,E)-2,4,6-Octatriene, showed a binding affinity of −3.8 kcal/mol and interacted with key residues including CYS103, ARG105, MET32, and VAL73.1,2,3,6-Tetrahydrobenzylalcohol, acetate, exhibited the highest binding affinity among all the ligands analyzed, with a value of −5.3 kcal/mol. It interacted with key residues such as TRP106, MET32, PRO76, CYS103, and TYR30. Ligand 12, Alloaromadendrene, showed a binding affinity of −6.9 kcal/mol and interacted with TRP208, CYS103, and TYR30. Phenol, 2-ethyl-4,5-dimethy, exhibited a binding affinity of −5.5 kcal/mol and interacted with key residues such as GLU31, TYR30, PRO76, and CYS103. Ligand 14, Phenol, 2-methyl-5-(1-methylethyl)-, showed a binding affinity of −5.6 kcal/mol and interacted with GLU31, HIS104, CYS103, and TYR30. Ligand 15, p-Cymen-7-ol, exhibited a binding affinity of −5.2 kcal/mol and interacted with key residues such as VAL29, TRP106, TYR30, and CYS103.1,5,5-Trimethyl-6-methylene-cyclohexene showed a binding affinity of −5.1 kcal/mol and interacted with key residues including PRO76, CYS103, and TYR30. Ligand 17, 1,3-Cyclohexadiene, 1-methyl-4-(1-methylethyl)-, exhibited a binding affinity of −5.0 kcal/mol and interacted with key residues such as CYS103, PRO76, TYR30, and ARG128. Finally, 2,6-Octadien-1-ol, 3,7-dimethyl-, acetate, (Z), exhibited a binding affinity of −5.2 kcal/mol and interacted with key residues such as GLU31, ARG105, ARG128, TRP208, and ARG105. Comparatively, the native ligand oxonic acid displayed a binding score of −4.8 kcal/mol, while the ligands Alloaromadendrene and Phenol, 2-methyl-5-(1-methyl ethyl)- exhibited notably stronger binding affinities of −6.9 and −5.6 kcal/mol, respectively, against the anti-microbial protein 1R4U.

## 4 Conclusion

The findings of this study underscore the unexplored potential of *E*. *globulus* leaves as a rich source of polyphenolic compounds endowed with notable antioxidant and antibacterial attributes. The essential oil derived from this plant emerges as a promising resource with potential health benefits, presenting a valuable avenue for combating various illnesses. A leading contribution of this study resides in the delineation of methodologies to formulate locally available and economically feasible disinfectants utilizing indigenous Pakistani flora. The integration of *E*. *globulus* aligns with the heightened demand for disinfectants amidst the COVID-19 pandemic, signifying its pertinence and timeliness. Furthermore, the molecular docking analysis augments the comprehension of intricate protein-ligand interactions, thereby furnishing a foundation for innovative therapeutic development. This study’s multifaceted insights offer a valuable reference for pharmacognosy or natural product researchers seeking to unearth novel therapeutic prospects from alternative perspectives. In summation, the present study’s outcomes substantiate *E*. *globulus* latent benefits, bridge the gap between traditional knowledge and contemporary healthcare needs, and facilitate a deeper exploration of potential therapeutic applications within the natural plant product domain.

## Data Availability

The original contributions presented in the study are included in the article/Supplementary Material, further inquiries can be directed to the corresponding authors.
