# Supplementary material for: Bioactive, antioxidant and antimicrobial properties of chemically fingerprinted essential oils extracted from Eucalyptus globulus: in-vitro and in-silico investigations
Source: Front Chem. 2023 Dec 8;11:1287317. doi: 10.3389/fchem.2023.1287317 (PMC10768562; doi:10.3389/fchem.2023.1287317)
Supplement: Supplementary file 1 [file DataSheet1.docx]

**Bioactive, Antioxidant and Antimicrobial Properties of chemically fingerprinted Essential Oil*s* extracted from *Eucalyptus globulus ssp.*: *In vitro* and *In silico* investigations**

**Supplementary File**


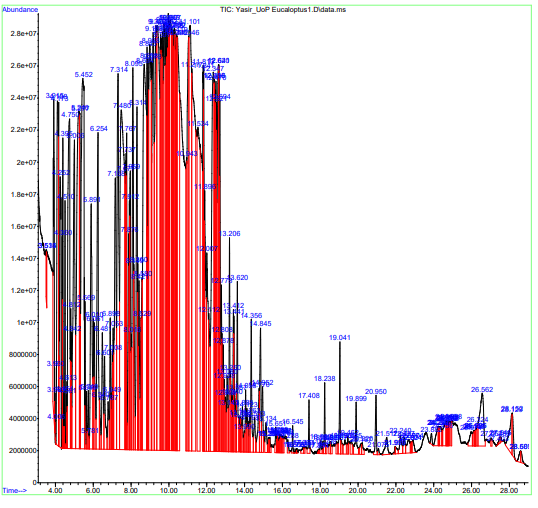


**Fig.S1:** GC-MS chromatogram of *Eucalyptus globulus* ssp. oil extract

**Table 5** Ligand Codes with 2D and 3D binding interactions with distance for 1AJ6.

| **Ligand Code** | **3D- Interactions** | **2D- Interactions** |
| --- | --- | --- |
| 1 | 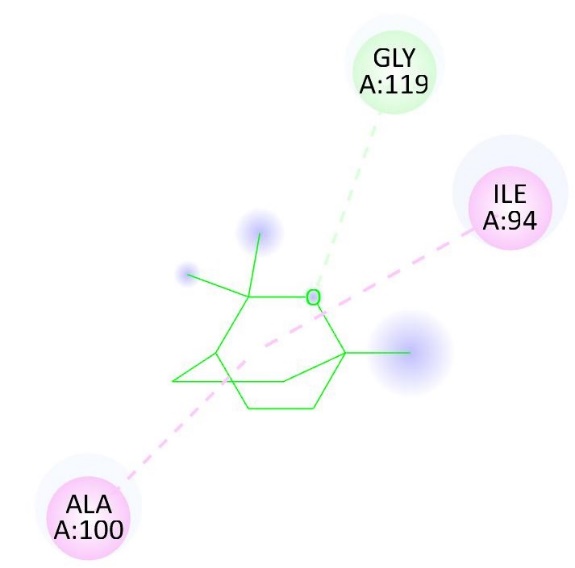 | 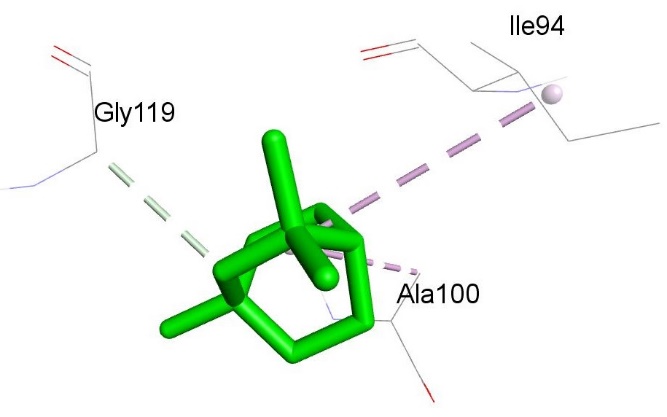 |
| 2 | 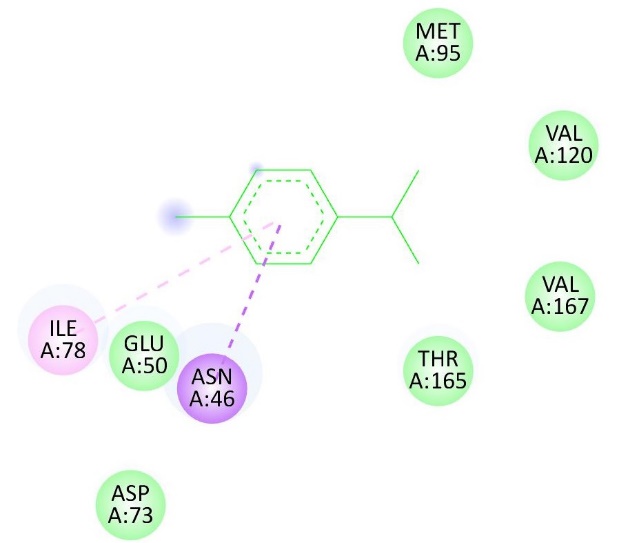 | 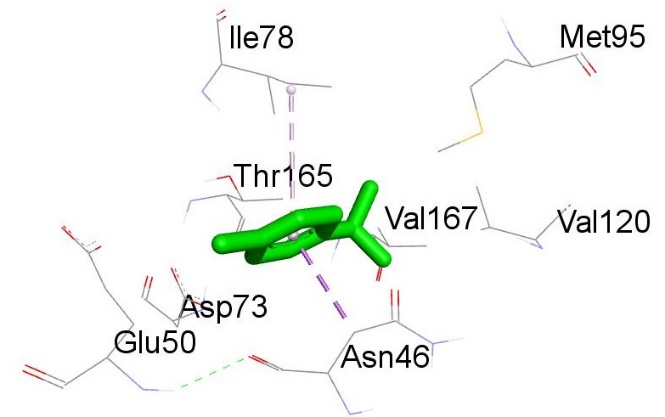 |
| 5 | 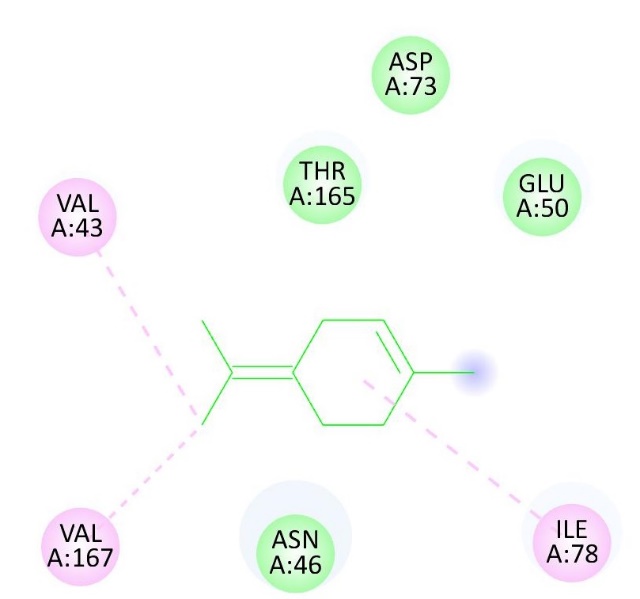 | 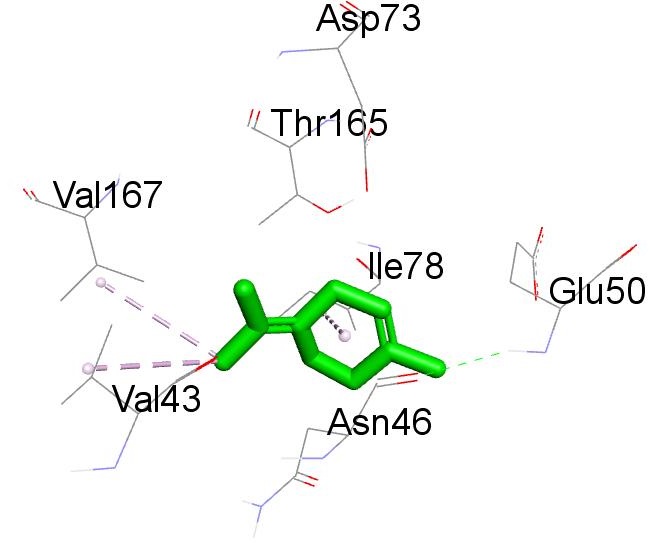 |
| 6 | 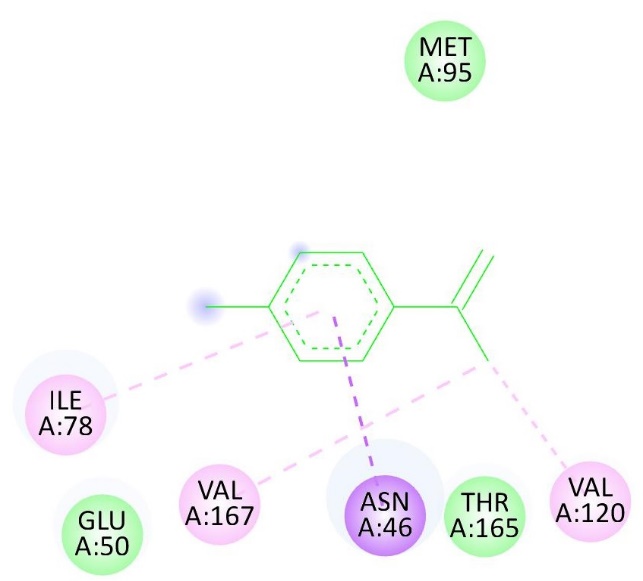 | 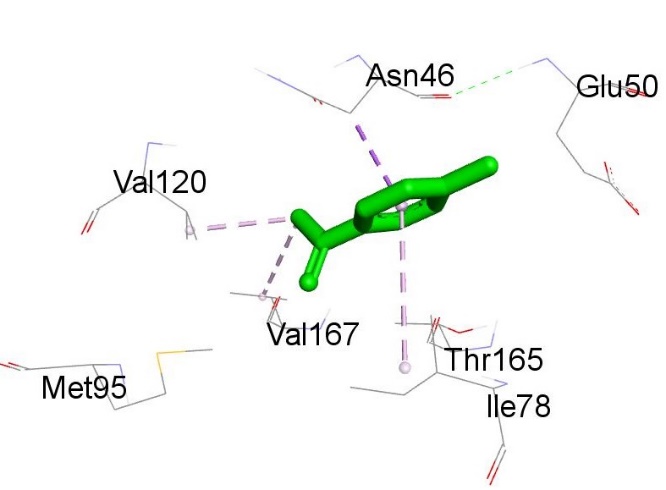 |
| 7 | 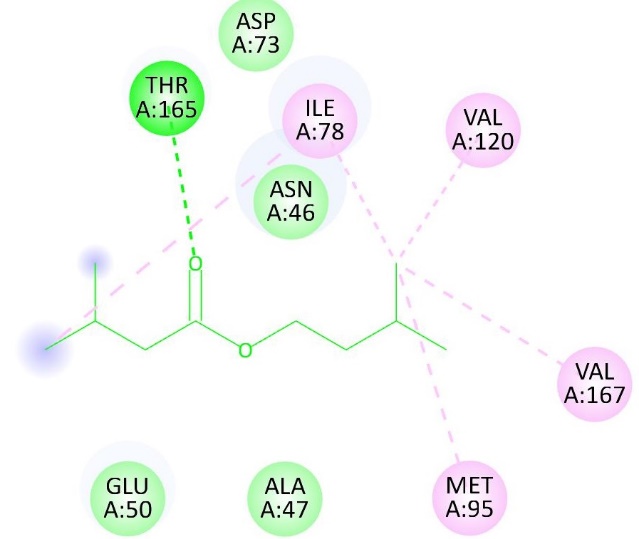 | 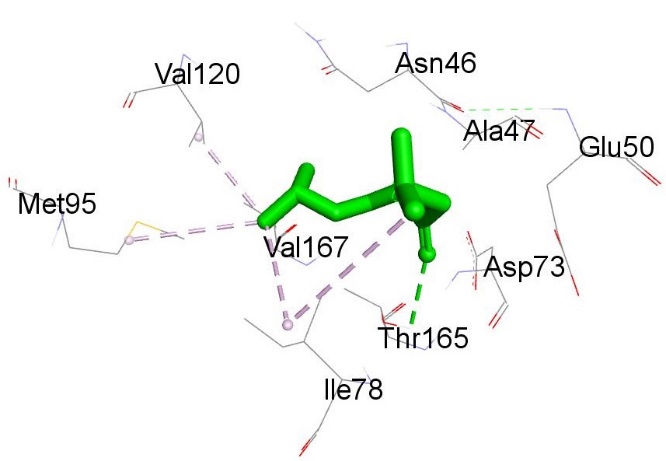 |
| 8 | 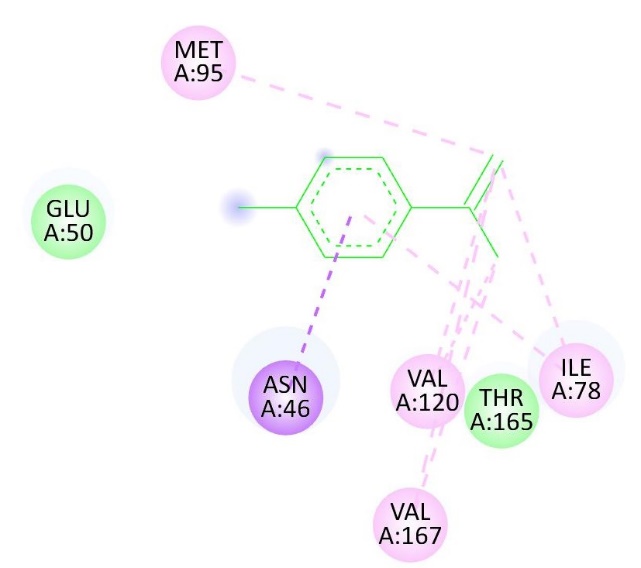 | 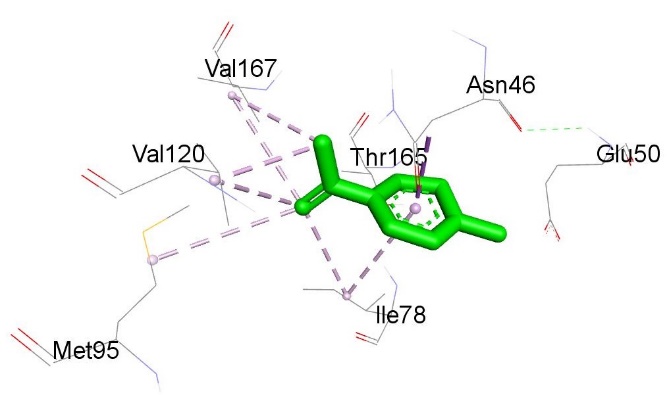 |
| 9 | 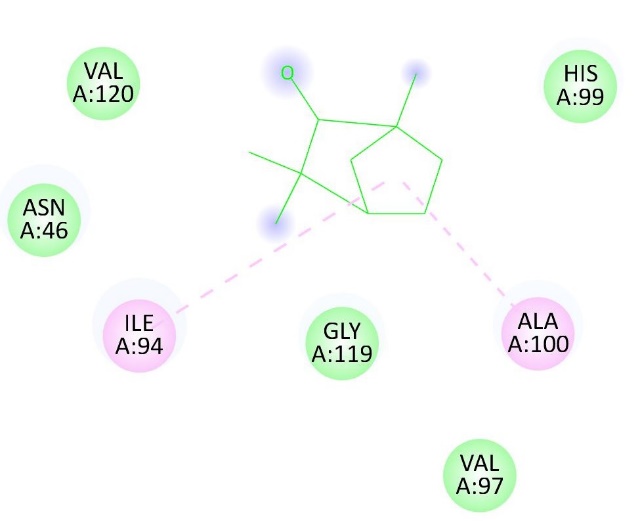 | 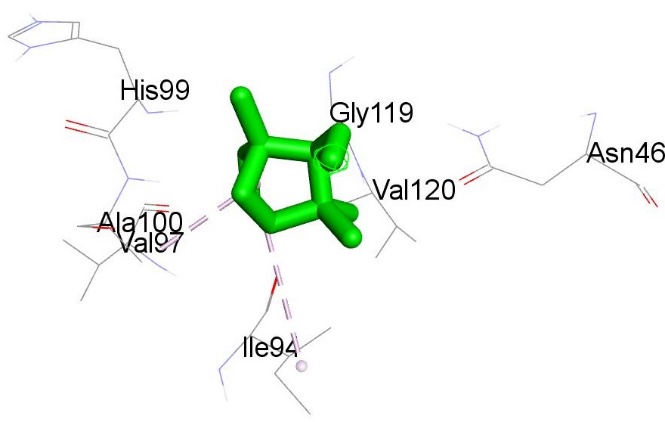 |
| 10 | 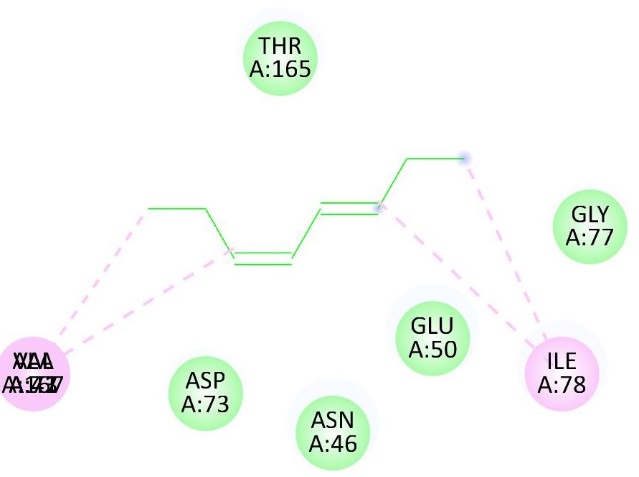 | 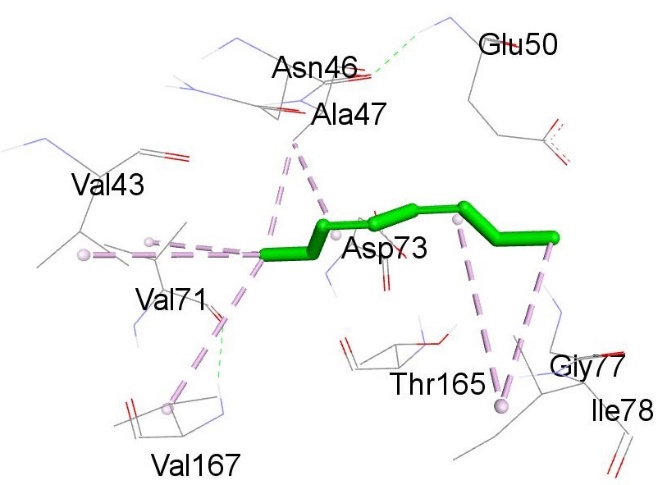 |
| 11 | 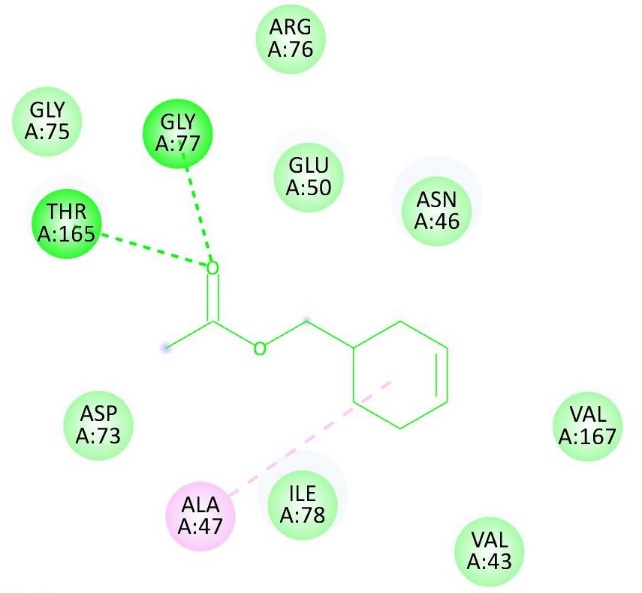 | 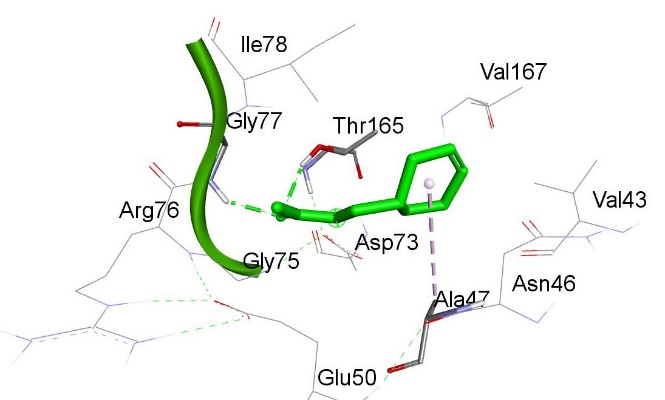 |
| 12 | 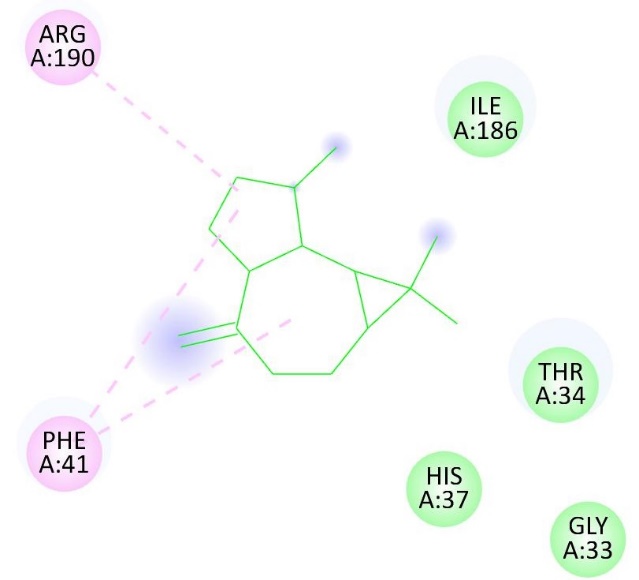 | 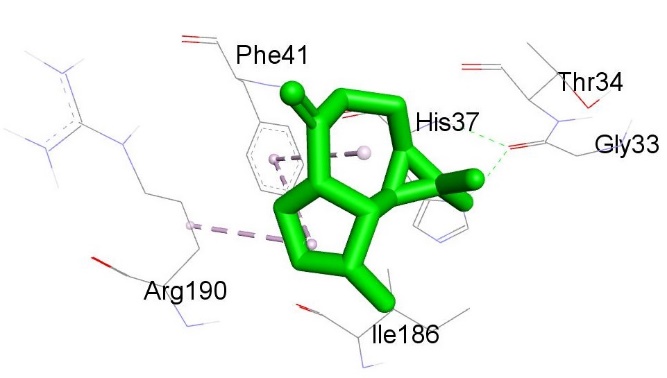 |
| 13 | 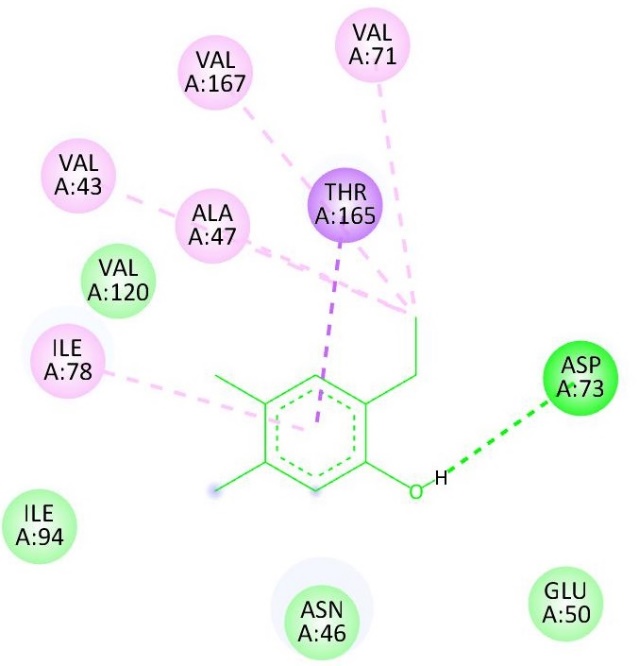 | 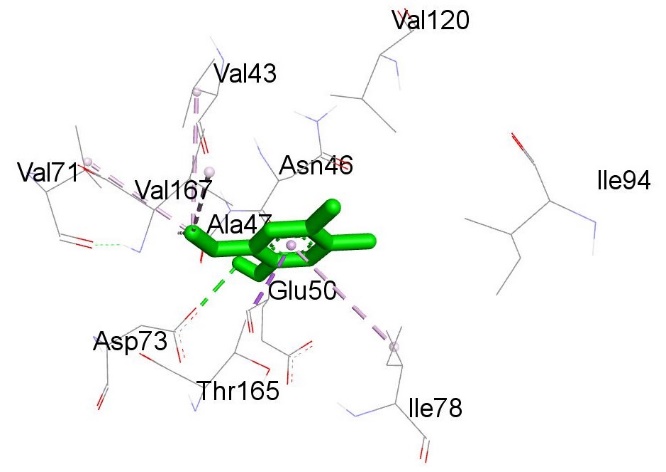 |
| 14 | 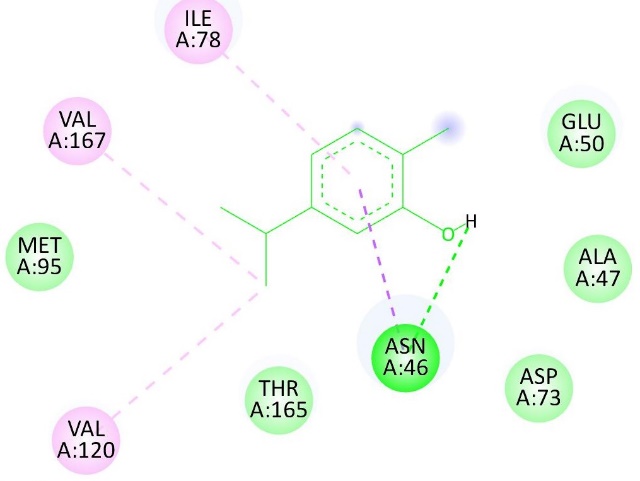 | 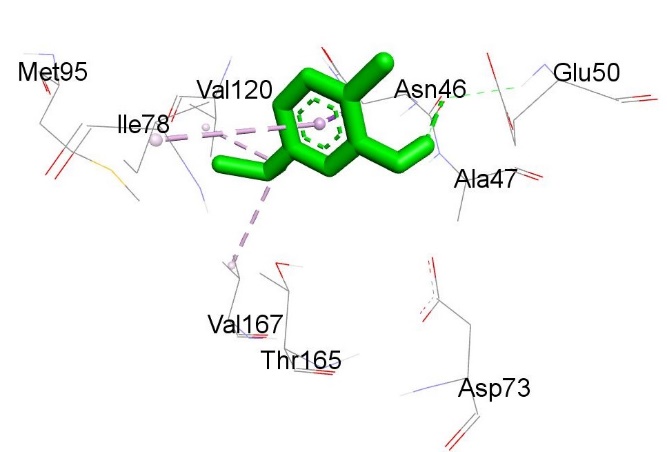 |
| 15 | 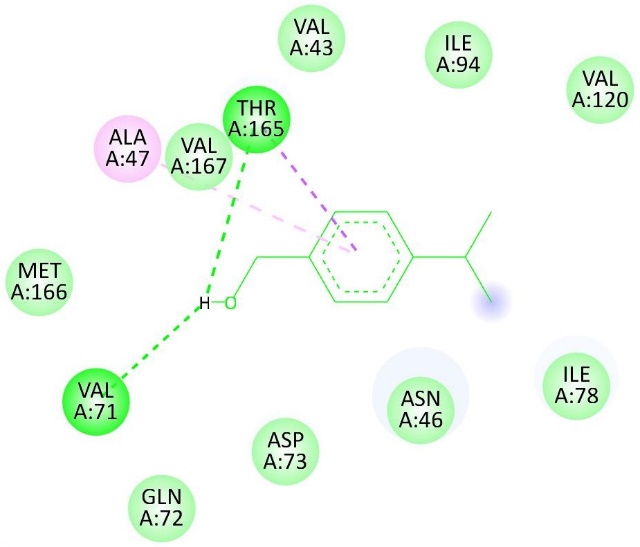 | 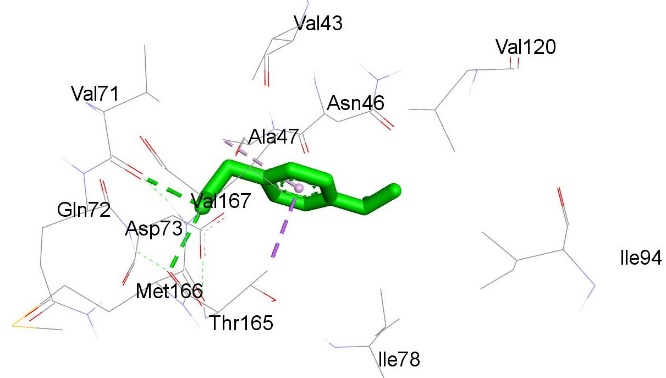 |
| 16 | 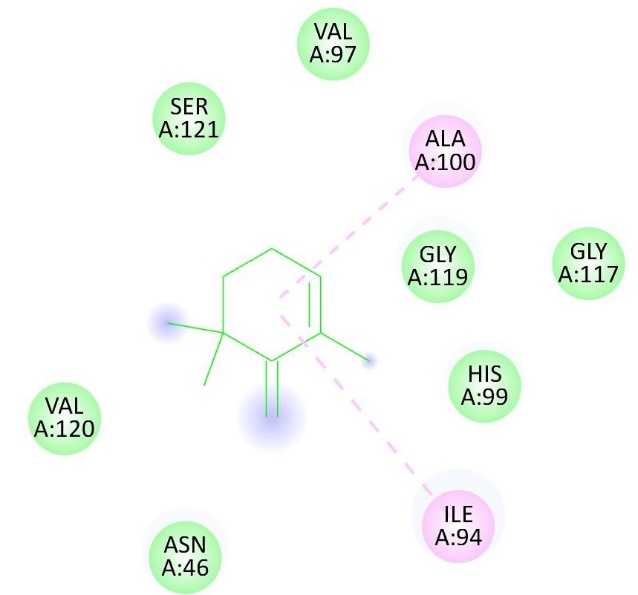 | 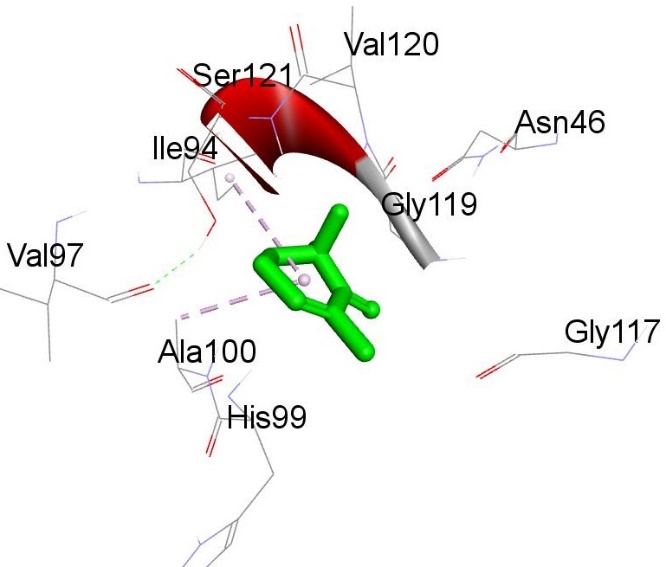 |
| 17 | 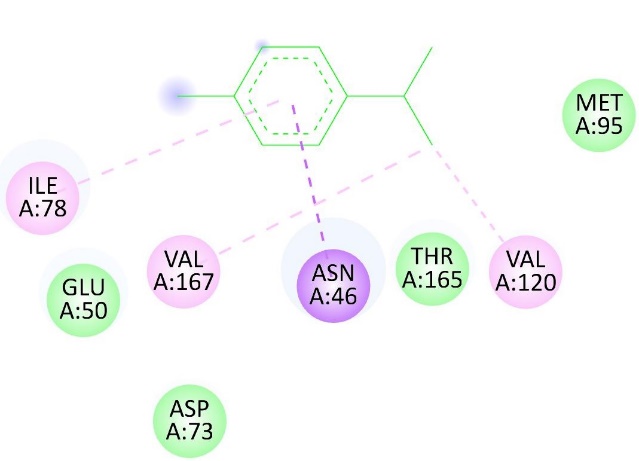 | 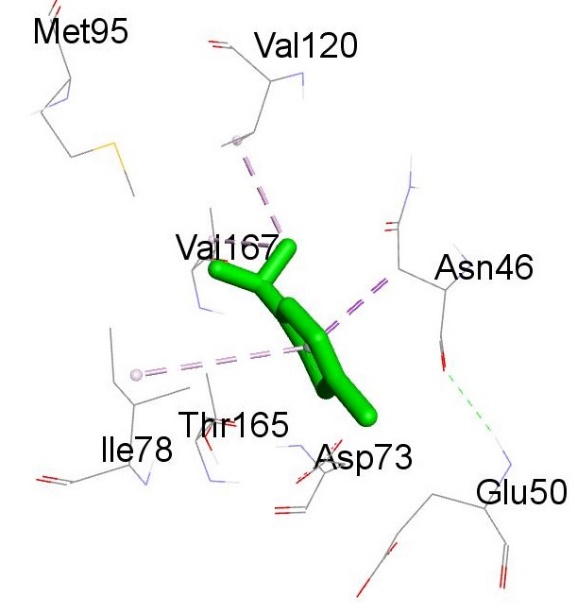 |
| 18 | 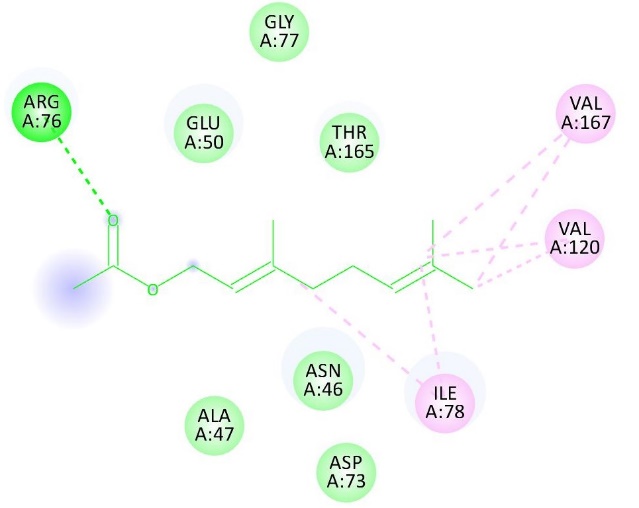 | 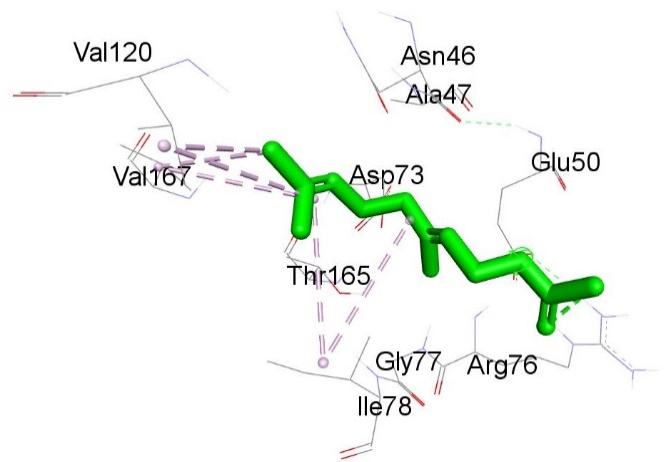 |

**Table 6.** Ligand Codes with 2D and 3D binding interactions with distance for 1R4U.

| **Ligand Code** | **3D- Interactions** | **2D- Interactions** |
| --- | --- | --- |
| 1 | 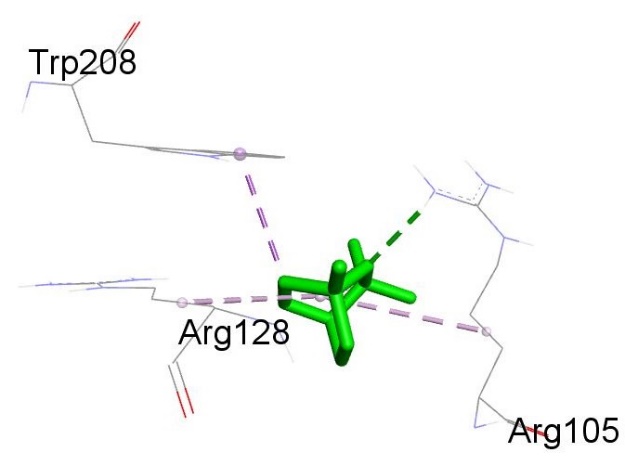 | 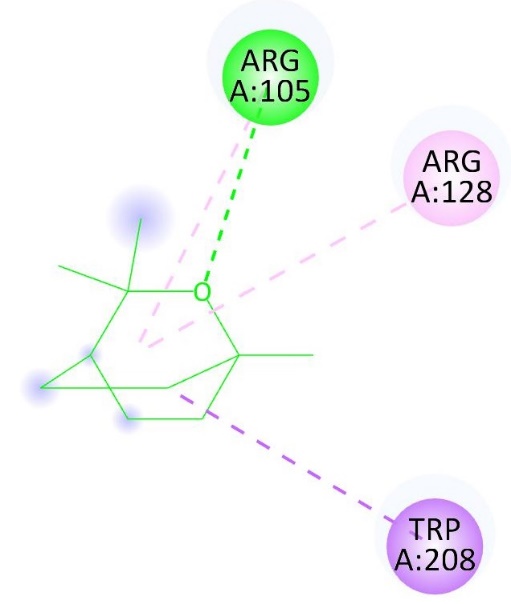 |
| 2 | 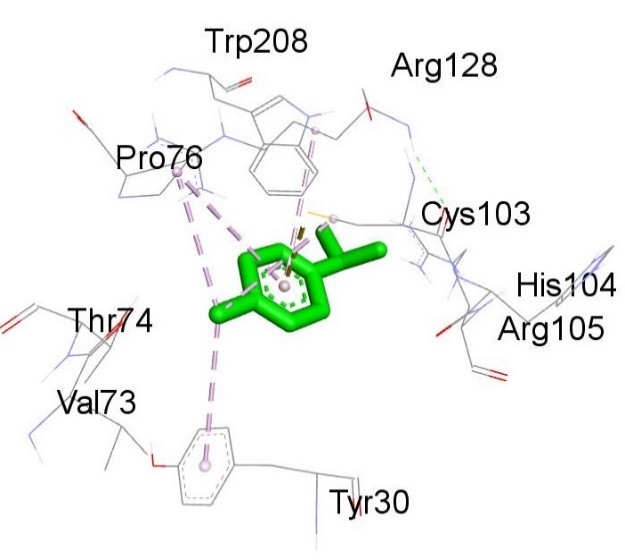 | 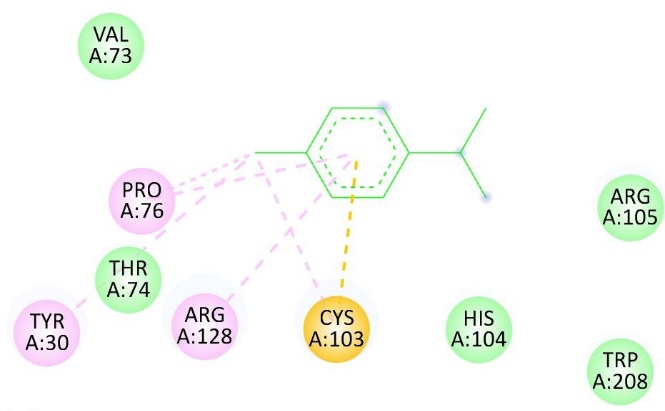 |
| 5 | 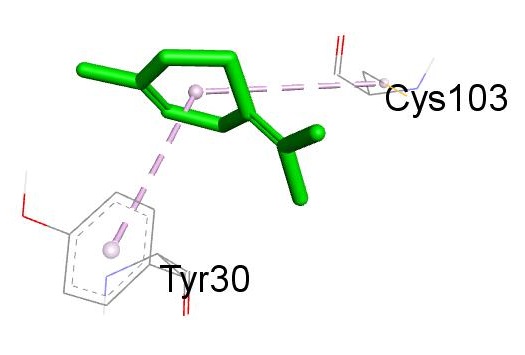 | 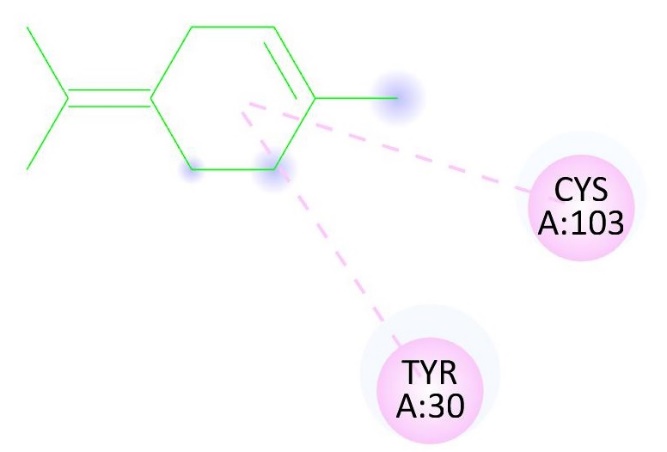 |
| 6 | 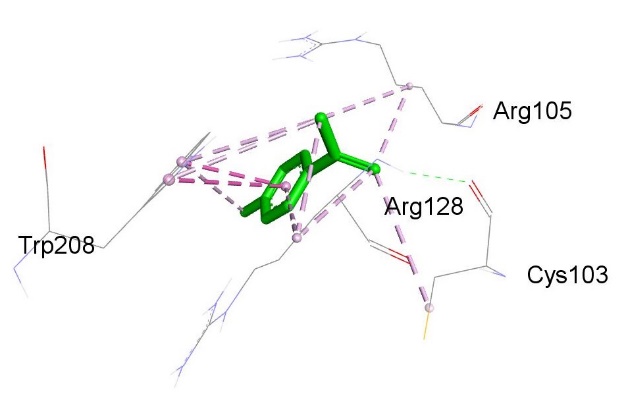 | 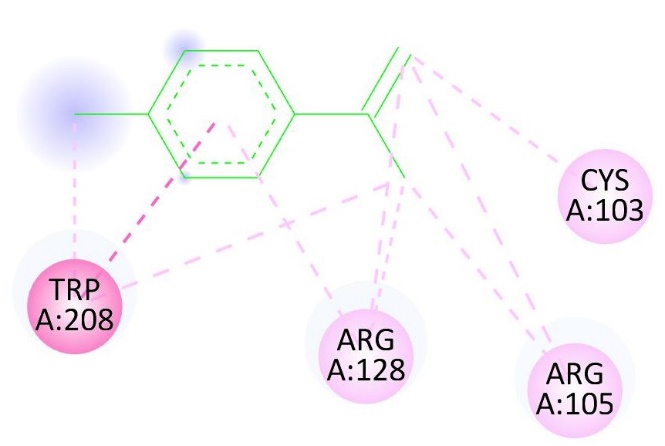 |
| 7 | 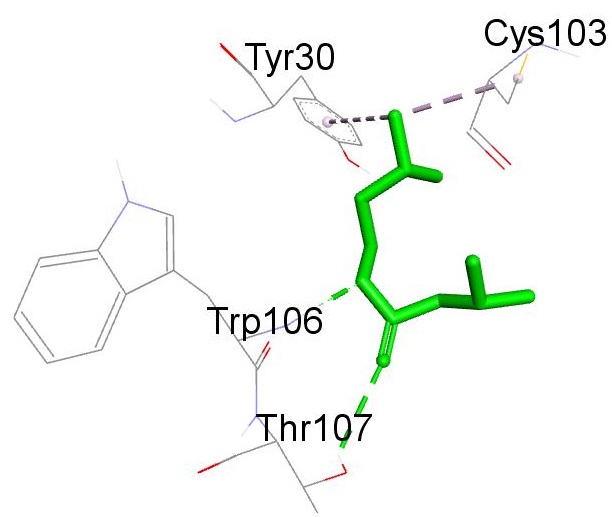 | 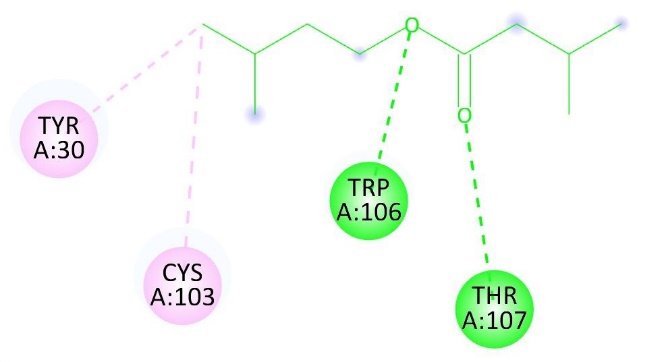 |
| 8 | 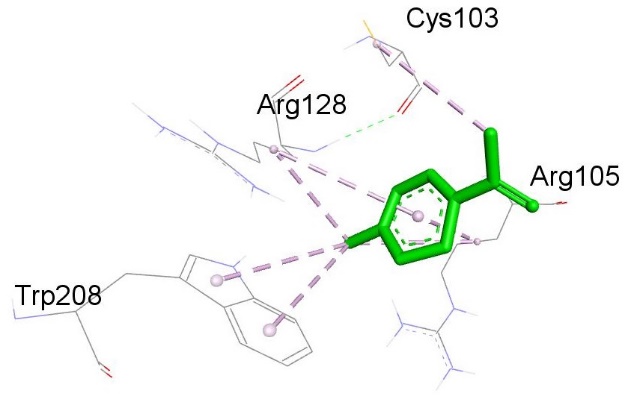 | 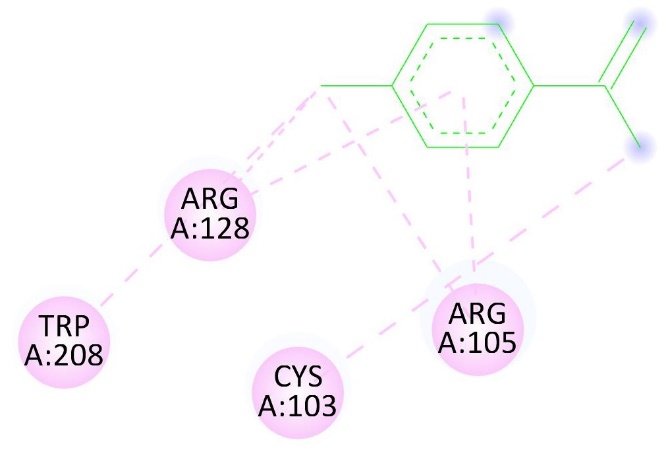 |
| 9 | 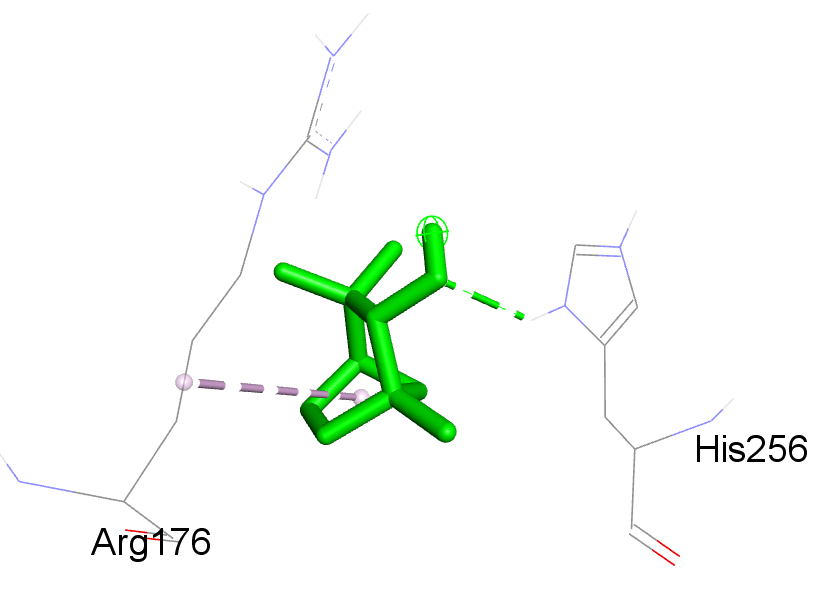 | 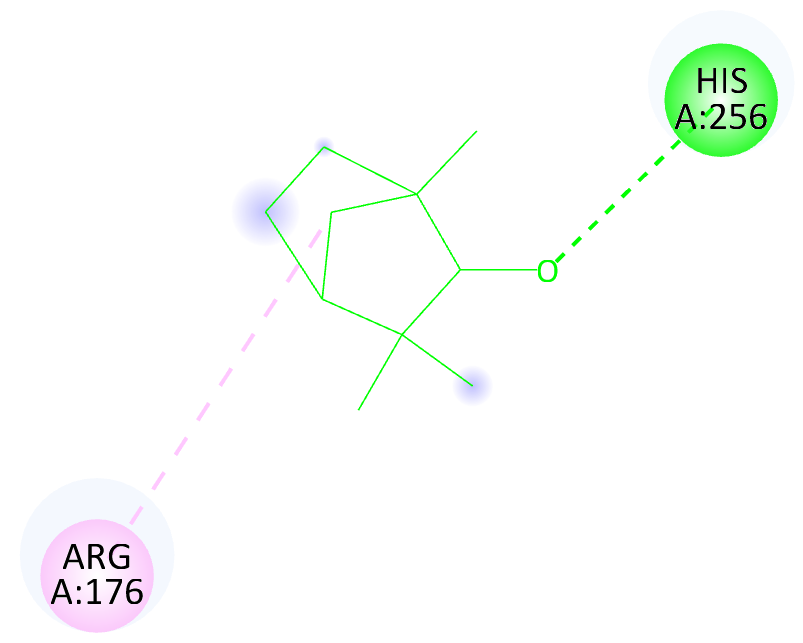 |
| 10 | 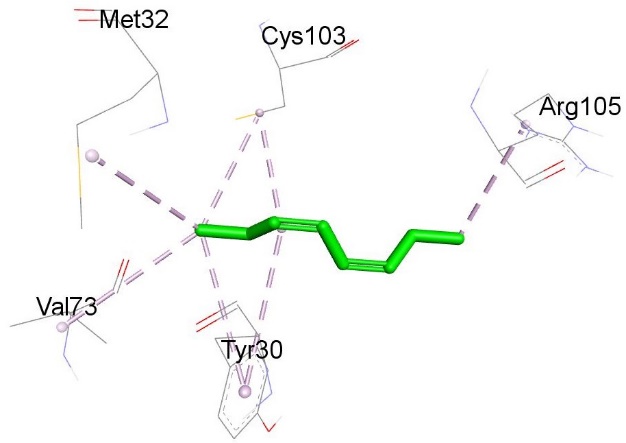 | 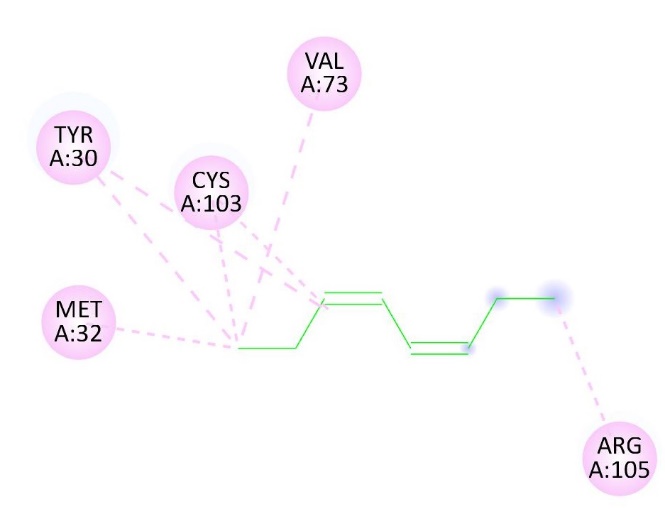 |
| 11 | 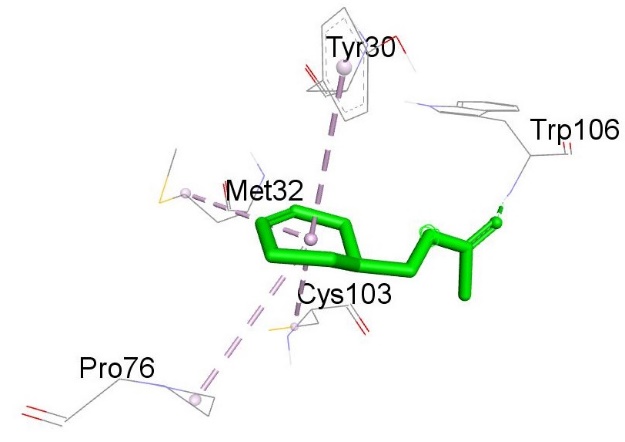 | 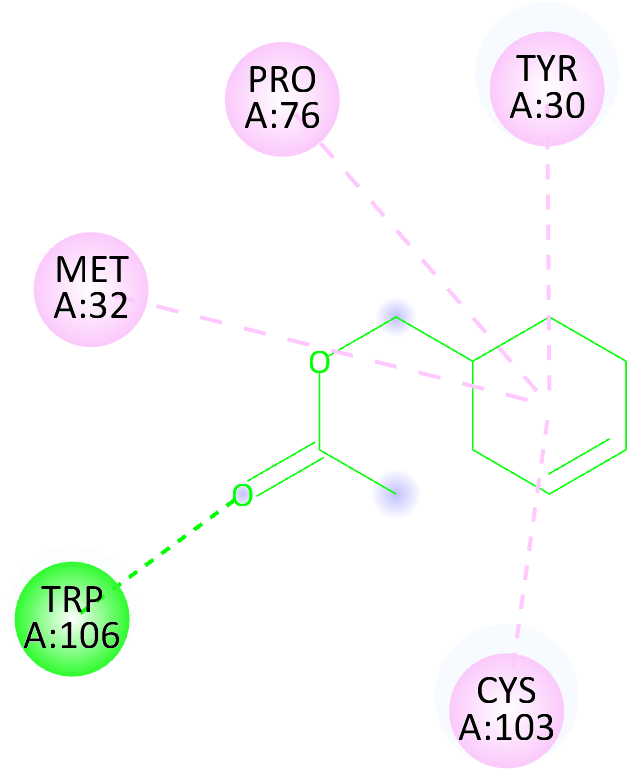 |
| 12 | 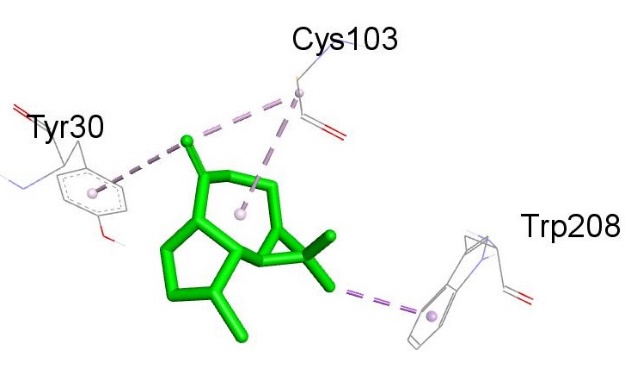 | 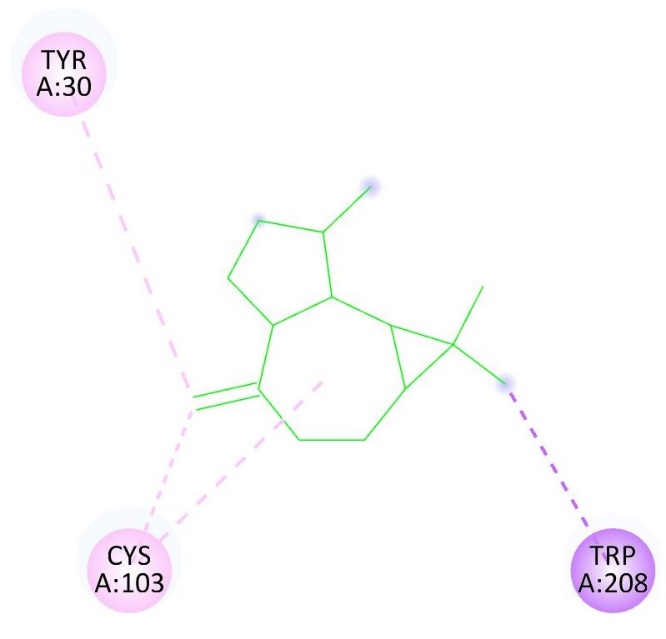 |
| 13 | 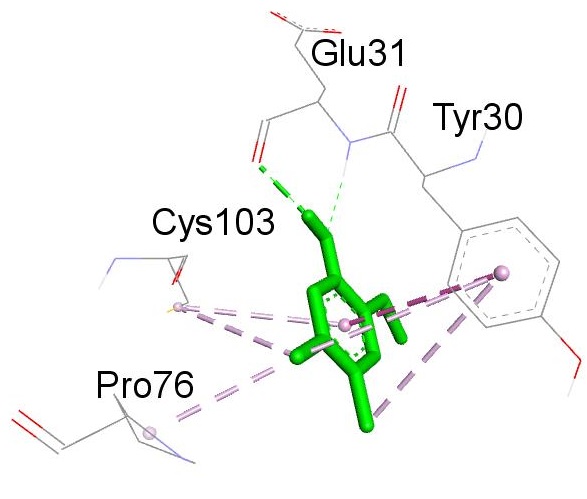 | 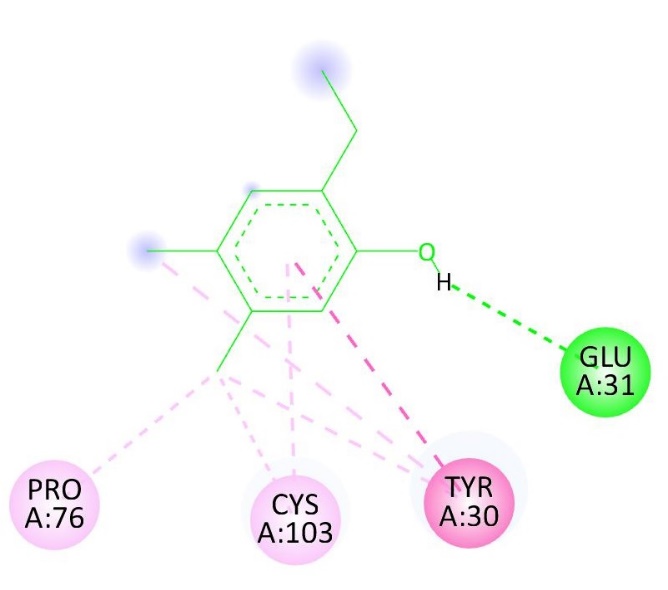 |
| 14 | 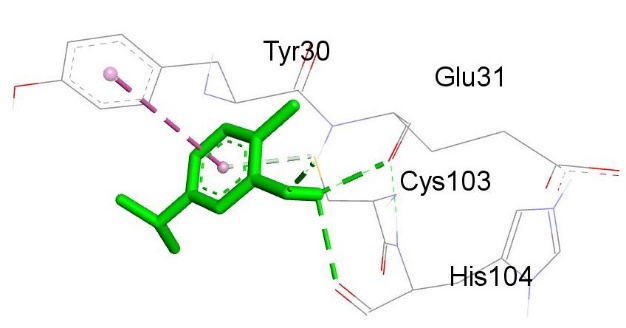 | 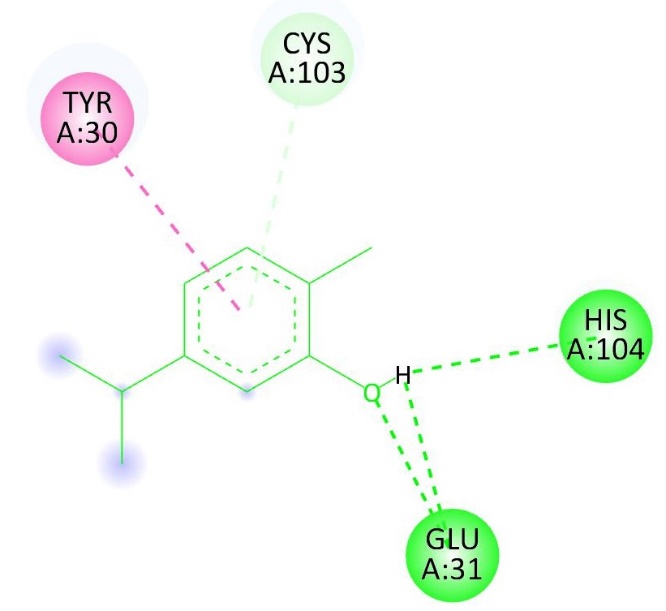 |
| 15 | 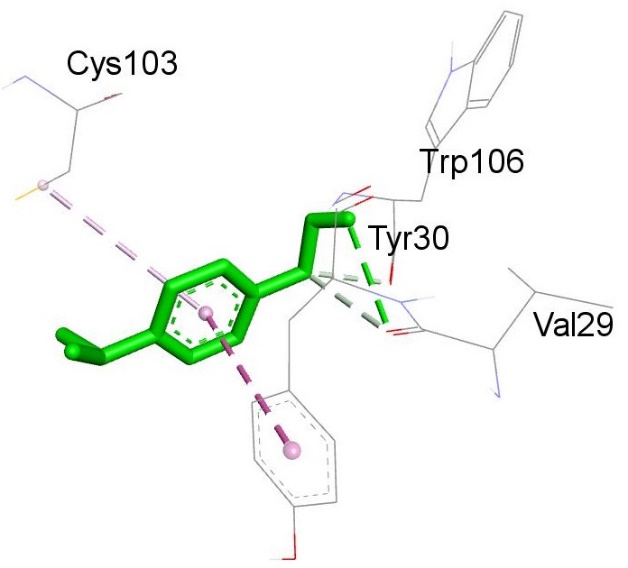 | 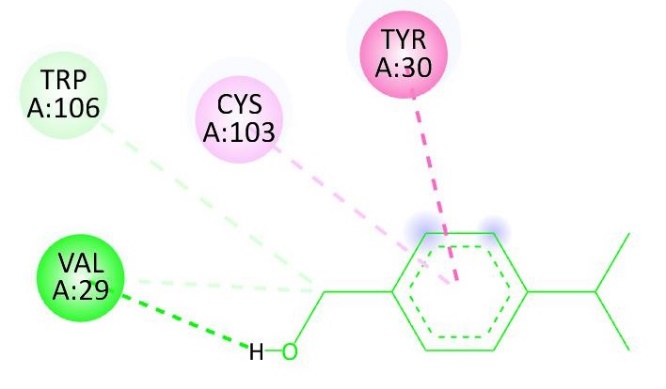 |
| 16 | 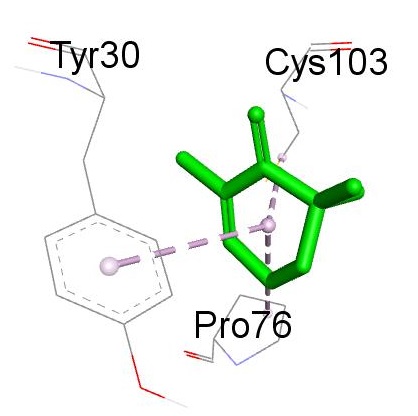 | 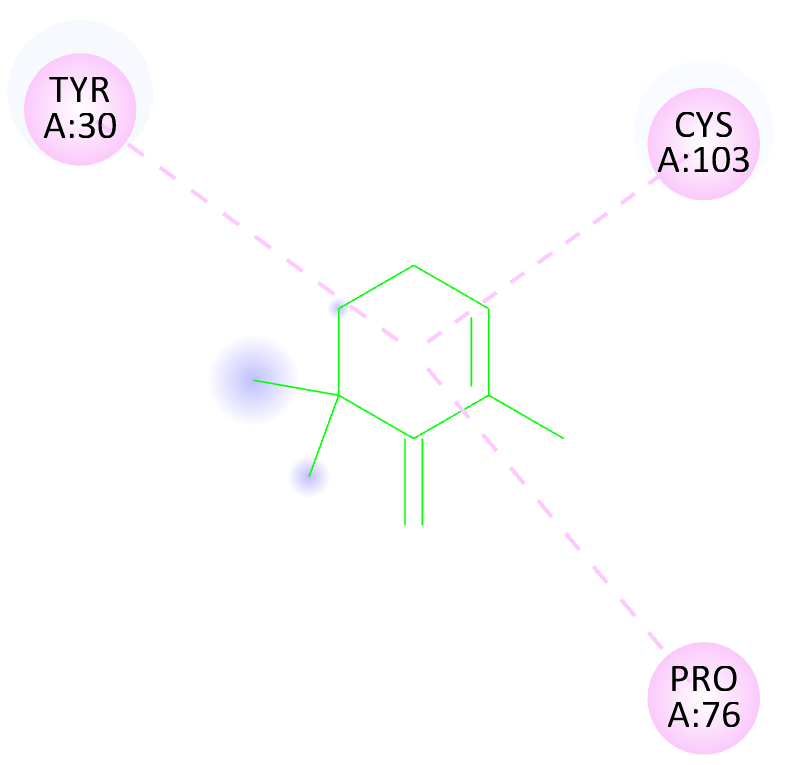 |
| 17 | 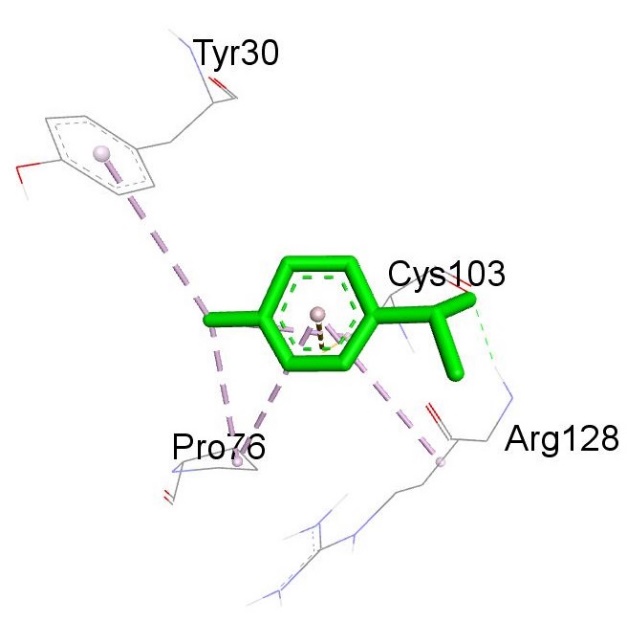 | 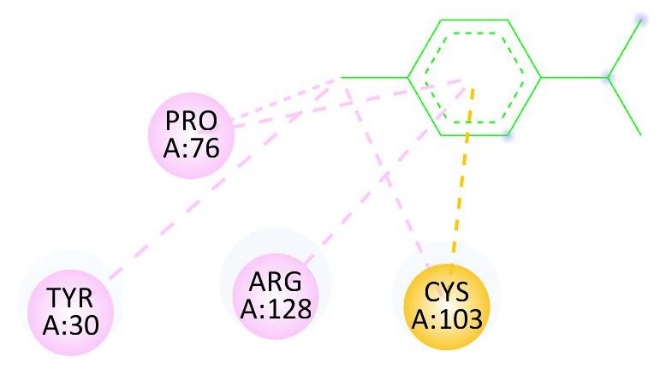 |
| 18 | 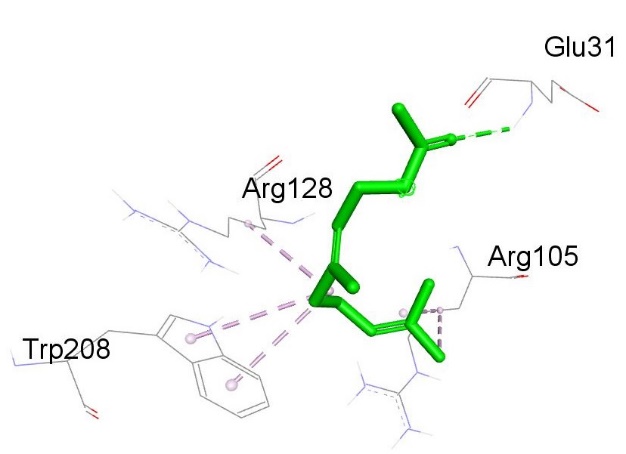 | 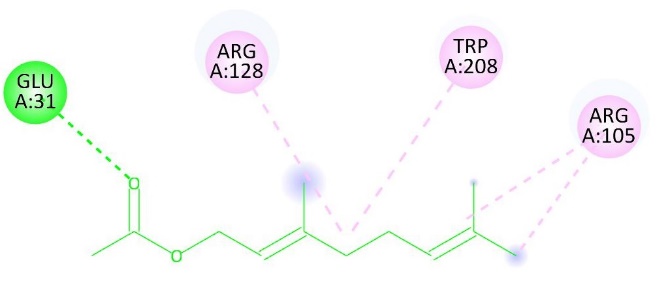 |
